# Stem Cell Review Series: Role of neurogenesis in age-related memory disorders

**DOI:** 10.1111/j.1474-9726.2008.00369.x

**Published:** 2008-08

**Authors:** Elodie Drapeau, Djoher Nora Abrous

**Affiliations:** 1 Doetsch's Laboratory, Columbia University, Department of PathologyP&S 14-511, 630 W 168th Street, New York, NY 10032, USA; 2 INSERM U862, Bordeaux Neuroscience Research Center, University of Bordeaux 2Bordeaux Cedex 33077, France

**Keywords:** aging, hippocampus, memory, neurogenesis, neuroplasticity, spatial learning

## Abstract

Neuroplasticity is characterized by growth and branching of dendrites, remodeling of synaptic contacts, and neurogenesis, thus allowing the brain to adapt to changes over time. It is maintained in adulthood but strongly repressed during aging. An age-related decline in neurogenesis is particularly pronounced in the two adult neurogenic areas, the subventricular zone and the dentate gyrus. This age-related decline seems to be attributable mainly to limited proliferation, associated with an age-dependent increase in quiescence and/or a lengthening of the cell cycle, and is closely dependent on environmental changes. Indeed, when triggered by appropriate signals, neurogenesis can be reactivated in senescent brains, thus confirming the idea that the age-related decrease in new neuron production is not an irreversible, cell-intrinsic process. The coevolution of neurogenesis and age-related memory deficits – especially regarding spatial memory – during senescence supports the idea that new neurons in the adult brain participate in memory processing, and that a reduction in the ability to generate new neurons contributes to the appearance of memory deficits with advanced age. Furthermore, the age-related changes in hippocampal plasticity and function are under environmental influences that can favor successful or pathological aging. A better understanding of the mechanisms that regulate neurogenesis is necessary to develop new therapeutic tools to cure or prevent the development of memory disorders that may appear during the course of aging in some individuals.

## Introduction

**Fig. 1:**
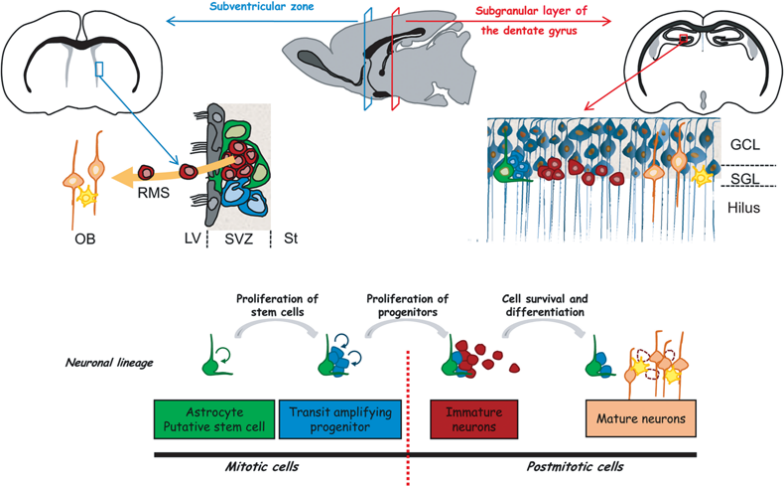
The main neurogenic areas of the adult brain. Adult neurogenesis has been described in the subventricular (SVZ)/olfactory bulb (OB) and in the hippocampal system. Representations of the different stages of adult neurogenesis that may be affected by aging; glial fibrillary acidic protein-positive astrocytes (green) have been identified as *in vivo* stem cells in the SVZ and stem-like cells in the dentate gyrus (DG). They divide slowly to give rise to transit amplifying progenitors (blue), which in turn generate immature cells (red) able to differentiate either into neurons (orange) or glial cells (yellow). A significant fraction of the newborn cells die during the maturation process (dotted cells). RMS, rostral migratory stream; LV, lateral ventricle; St, striatum; GCL, granule cell layer; SGL, subgranular layer.

The vast majority of cells in the adult central nervous system (CNS) are generated during the embryonic and early postnatal period. However, it is now well accepted that new neurons are continuously added in specific regions of the mammalian brain throughout adulthood. In rodents, monkeys, and humans, neurogenesis has been described within the hippocampal formation (HF) in the subgranular layer (SGL) of the dentate gyrus (DG), and in the subventricular zone (SVZ) (Gross, 2000; Abrous *etal*., 2005; Ming & Song, 2005; Christie & Cameron, 2006; Lledo *etal*., 2006; Scharfman & Hen, 2007) (Fig. 1).

The process of neurogenesis in the young adult brain can be divided into a series of distinct developmental steps, which can be examined separately and include the proliferation of precursor cells, the survival of newly born cells, the migration of these cells, and, finally, their differentiation into mature functional neurons. Precursor cells can be either *stem cells*, characterized by a slow-dividing cell cycle, long-term self-renewal potential, and multipotentiality, or *progenitors*, which exhibit a higher dividing rate of turnover and reduced self-renewal abilities. Progenitors are more differentiated than stem cells, and their multipotentiality is still a matter of debate.

In the DG, new neurons originate from cells located in the SGL at the border of the granule cell layer (GCL) facing hilus. These cells are slowly proliferating astrocytes [identified on the basis of their expression of glial fibrillary acidic protein (GFAP) and their ultrastructural properties], presumably radial glia (Garcia *etal*., 2004b; Seri *etal*., 2004). However, their stem cell nature is still a matter of debate as *in vitro* studies have shown that cells isolated specifically from the DG have only limited self-renewal abilities and are restricted to the neuronal lineage (Seaberg & van der Kooy, 2002). This lack of stem cell properties may be caused by the experimental *in vitro* conditions, yet we will refer to these cells as ‘stem-like’ cells rather than stem cells. The SGL-dividing astrocytes generate immature, GFAP-negative, intermediate-amplifying precursors that in turn divide. Their daughter cells migrate a short distance into the GCL. A significant fraction of the newly generated neuronal cells undergo programmed cell death (Gould *etal*., 1999; Sun *etal*., 2004; Dupret *etal*., 2007), while the surviving cells successfully differentiate, mostly into hippocampal granule cells, extending axons into the CA3 region (Stanfield & Trice, 1988; Hastings & Gould, 1999; Markakis & Gage, 1999; van Praag *etal*., 2002) and becoming functionally integrated in the hippocampal circuitry (Hastings & Gould, 1999; Carlen *etal*., 2002; van Praag *etal*., 2002; Jessberger & Kempermann, 2003; Kee *etal*., 2007; Toni *etal*., 2007).

In the SVZ, GFAP cells have been identified as stem cells (Doetsch *etal*., 1999a). They divide slowly to generate rapidly dividing transit amplifying cells, which in turn give rise to neuroblasts. These neuronal precursors born in the SVZ migrate tangentially along the rostral extension of the SVZ toward the olfactory bulb (OB), constituting the rostral migratory stream (RMS). As in the DG, a high proportion of the cells generated in the SVZ die after birth (Petreanu & Alvarez-Buylla, 2002; Winner *etal*., 2002). After reaching the core of the OB, surviving cells move radially into the granular and periglomerular layers where they differentiate into functional interneurons (Luskin, 1993; Lois & Alvarez-Buylla, 1994; Carlen *etal*., 2002; Huang & Bittman, 2002; Magavi *etal*., 2005).

Although the adult brain retains remarkable plastic capabilities, aging is classically associated with a decline in several forms of neuronal plasticity. Indeed, the ability of neurons to modify their connectivity by changing their number and spine shape in response to various environmental (e.g. brain damage) and physiological stimuli is less robust in the senescent brain (see, for review, Petit & Ivy, 1988; Burke & Barnes, 2006). In this context, it is logical that neurogenesis, which continues throughout life, decreases in both of the neurogenic areas with increasing age.

Even if the functional significance of this ongoing adult neurogenesis is not fully understood, evidence has been provided that newly produced neurons play an important role in functions associated with the neurogenic areas. Adult neurogenesis has been shown to be involved in several brain functions (e.g. memory and emotion) and pathologies (e.g. depression and addiction) (Abrous *etal*., 2005). Here, we will focus mainly on neurogenesis and memory. Indeed, the functional consequences of the age-related decline in neurogenesis have been examined only in relation to this function, known to be altered in the course of aging (Stevens & Cain, 1987; Grady & Craik, 2000; Hulshoff Pol *etal*., 2000; Kaneda *etal*., 2000). Briefly, in young subjects, an increasing number of studies have correlated changes in the rate of dentate neurogenesis with spatial memory ability, and changes in adult-born olfactory neurons to olfactory memory (Leuner *etal*., 2006; Lledo *etal*., 2006; Abrous & Wojtowicz, 2008). Based on these observations, it has been hypothesized that the age-related decline in neurogenesis, together with a decline in other type of structural and synaptic plasticity, may contribute to the normal physiological reduction in memory function associated with aging.

Consequently, in this review, we propose an overview of the current knowledge about the evolution of adult neurogenesis during aging and its functional consequences. We will first describe the changes in neurogenesis observed during aging and then focus on ‘intrinsic’ and extracellular mechanisms (i.e. the changes in the environmental niche for neurogenesis) that can explain the observed age-related modifications of neuronal production. Finally, we will focus on the relationship between the age-related decline in neurogenesis and age-dependent memory impairments, and discuss the possible role of decreased neurogenesis in the memory decline reported in some senescent subjects.

## Neurogenesis in the aging brain

Multiple reports demonstrate that the production of new neurons in neurogenic brain regions declines dramatically with age. However, in most of these studies, neurogenesis was measured by labeling the newborn cells with a thymidine analogue such as bromodeoxyuridine (BrdU) or by expression of markers for proliferation and immature cells. Various BrdU dosages, injection frequencies, and survival times have been used, yielding different results concerning fate or numbers of newborn cells, and making it difficult to compare these studies in terms of absolute numbers. Moreover, the basal level of neurogenesis, the onset of its age-related down-regulation, and ‘how old is old’ vary significantly among species, strains (and median lifespan), sex, and experimental conditions. Consequently, all of these variables could explain the discrepancies between experiments that we will report.

**Fig. 2:**
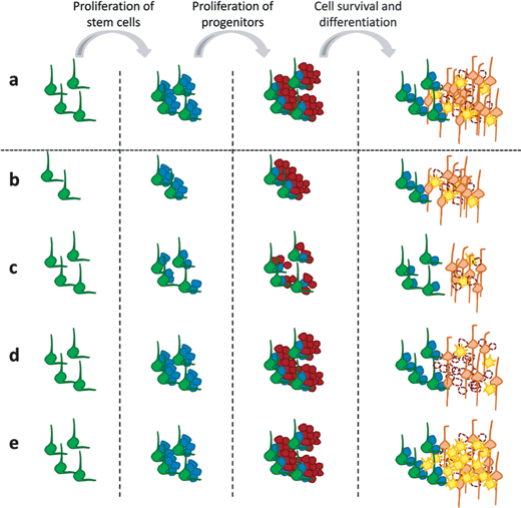
Different stages of adult neurogenesis and their potential modifications during aging. Neurogenesis in young adult (a) and aged (b–e) brain. The decline of neurogenesis observed during aging can be the consequence of: (b) a decrease of the number of precursors; (c) a reduction in the proliferative activity of the precursors as a consequence either of a lengthening of the cell cycle or an increase in their quiescence; (d) a decrease of the proportion of newly generated cells surviving after the first weeks of maturation; (e) a differentiation biased toward a glial phenotype. It is likely that a combination of all of these mechanisms is involved in the aging of neurogenic areas.

In the following section, we will review the effect of aging on the different steps leading to the production of new neurons. Neurogenesis in the adult brain can be divided into three phases in accordance with the sequence of neurogenesis during development: (i) proliferation, when new cells are generated; (ii) survival of a portion of these new cells and their migration toward target areas; and (iii) terminal differentiation into a neuronal or glial phenotype. Substantial changes in some or all of the above events may underlie the reduction in hippocampal neurogenesis during aging (Fig. 2). The aging process could either deplete the number of precursors or alter their mitotic activity; both would ultimately lead to a reduction in the actual number of newly born cells. Additionally, the newborn cells could die before they differentiate into granule neurons or toward another cell phenotype.

### In the DG

The time course of ***cell proliferation*** has been extensively studied in rodents (see Table 1) using injected proliferation markers such as BrdU and tritiated thymidine associated with short survival periods (usually 24 h after the injection as this is sufficient for a newborn cell to complete at least one cell cycle) to label proliferating cells and their progeny. Alternatively, cell proliferation may be studied using intrinsic proliferation markers such as Ki67, proliferating cell nuclear antigen (CNA), the phosphorylated histone 3 (HH3), and the minichromosome maintenance deficient 2 mitotin (MCM-2) (Maslov *etal*., 2004) that are transiently expressed by cycling cells. All are considered to be reliable markers to assay the proliferative activity of precursors (Kee *etal*., 2002; Wojtowicz & Kee, 2006; Taupin, 2007). In the rat DG, cell proliferation reaches a peak during the second postnatal week (Schlessinger *etal*., 1975), and then declines dramatically with increasing age. In a pseudo-longitudinal study, cell proliferation was reported to decrease by 80% from adolescence (28 days) to adulthood (3 months), by 70% from adulthood to middle age (12 months) and by 60% from middle age to senescence (22 months) (Lemaire *etal*., 2000). Although many studies found an overall age-related decline in cell proliferation (Kuhn *etal*., 1996; Kempermann *etal*., 1998b; Cameron & McKay, 1999; Lemaire *etal*., 2006; Molofsky *etal*., 2006), the existence of a decline between middle age and senescence is still a matter of debate because some studies reported a significant effect (Bizon & Gallagher, 2003; Bondolfi *etal*., 2004; Rao *etal*., 2006) while others did not (Seki & Arai, 1995; Lichtenwalner *etal*., 2001; Nacher *etal*., 2003; Heine *etal*., 2004a; Rao *etal*., 2005; Driscoll *etal*., 2006; Kronenberg *etal*., 2006). Notably, BrdU-labeled precursors appeared to be lost equally at rostral and caudal levels, as well as in suprapyramidal and infrapyramidal blades of the GCL (Olariu *etal*., 2007). In contrast, cell proliferation in the hilus was not at all (Kuhn *etal*., 1996) or only slightly (Lichtenwalner *etal*., 2001) affected by aging, making the age-related decrease in proliferation specific to the GCL.

**Table 1:** Influence of aging on cell proliferation within the subventricular zone (SVZ) and the dentate gyrus (DG)

Proliferation						

Reference	Species	Strains	Sex	Ages	Area	Results
(Bizon & Gallagher, 2003)	Rat	Long-Evans	Male	7, 13, 25 months	DG	#BrdU cells: 7 m > 13 m > 25 m
(Bondolfi *etal*., 2004)	Mouse	C57BL/6	Male	2, 12, 18 months	DG	#BrdU cells: 2 m > 12 m > 18 m
(Brunson *etal*., 2005)	Rat	SD	Male	3 and 12 months	DG	#BrdU cells: 3 m > 12 m
(Cameron & McKay, 1999)	Rat	SD	?	5 and 26 months	DG	#BrdU cells: 5 m > 25 m
(Cuppini *etal*., 2006)	Rat	SD	Male	2, 5, 12 months	DG	#BrdU cells: 2 m > 5 m > 12 m
(Driscoll *etal*., 2006)	Rat	FBNF1	Female	3, 12, 24 months	DG	#Ki67 cells: 3 m > 12 m = 24 m
(Enwere *etal*., 2004)	Mouse	C57BL/6	Male	2 and 24 months	SVZ	#BrdU, Ki67, Mash cells: 2 m > 24 m
					RMS	#BrdU cells: 2 m > 24 m
(Gould *etal*., 1998)	Macaque	*M. mulatta* and *fascicularis*	Male and female	5, 7–16, 23 years	DG	#BrdU cells: 5y > 7–16y > 23y
(Hattiangady & Shetty, 2008)	Rat	F344	Male	4, 12, 24 months	DG	%Sox 2/BrdU cells: 15 > 2.5 > 1%Sox 2/Ki67 cells: 25 > 8 > 4
(Heine *etal*., 2004a)	Rat	Wistar	Male	2 weeks, 6 weeks, 12, 24 months	DG	#BrdU cells: 2w > 6w > 12 m = 24 m
(Kempermann *etal*., 1998b)	Mouse	C57BL/6	Female	6 and 18 months	DG	#BrdU cells: 6 m > 18 m
(Kim *etal*., 2004)	Rat	SD	Male	1, 2, 14 months	DG	#BrdU cells: 1 m > 2 m > 14 m
(Kronenberg *etal*., 2006)	Mouse	C57BL/6	Male	1.5, 9, 12, 24 months	DG	#BrdU cells: 1.5 m > 9 m > 12 m = 24 m
(Kuhn *etal*., 1996)	Rat	F344	Female	6 and 21 months	DG	#BrdU cells: 6 m > 21 m
					SVZ	#BrdU cells: 6 m = 21 m
(Lemaire *etal*., 2000)	Rat	SD	Male	1, 3, 10, 22 months	DG	#BrdU cells: 1 m > 3 m > 10 m > 22 m
(Lemaire *etal*., 2006)	Rat	Wistar	Male	4 and 26 months	DG	#BrdU cells: 4 m > 26 m#Ki67 cells: 4 m > 26 m
(Lichtenwalner *etal*., 2001)	Rat	BNxF344	Male	5, 18, 28 months	DG	#BrdU cells: 5 m > 18 m = 28 m
(Luo *etal*., 2006)	Mouse	?	?	2, 10, 22 months	SVZ	#BrdU cells: 2 > 22
(Maslov *etal*., 2004)	Mouse	C57BL/6	Male	4 and 26 months	SVZ	#BrdU cells: 4 m > 26 m
(McDonald & Wojtowicz, 2005)	Rat	SD	Male	1 and 12 months	DG	#BrdU cells: 1 m > 12 m
(Molofsky *etal*., 2006)	Mouse	C57BL/6	Male	2 and 24 months	SVZ	#BrdU cells: 2 m > 24 m
(Nacher *etal*., 2003)	Rat	F344	Female	3, 10, 20 months	DG	#BrdU cells: 3 m > 10 m = 20 m
(Olariu *etal*., 2007)	Rat	SD	Male	2.5 and 10 months	DG	#BrdU cells: 2.5 > 10 m#PCNA cells: 2.5 > 10 m
(Rao *etal*., 2005)	Rat	F344	Male	4, 12, 24 months	DG	#BrdU cells: 4 m > 12 m = 24 m
(Rao *etal*., 2006)	Rat	F344	Male	4, 12, 24 months	DG	#BrdU cells: 4 m > 12 m = 24 m#Ki67 cells: 4 m > 12 m > 24 m
(Seki & Arai, 1995)	Rat	Wistar	Male	1, 2, 4, 6, 12, 18 months	DG	#BrdU cells: 1 m > 2 m > 2 m > 4 m > 6 m > 12 m = 18 m
(Simon *etal*., 2005)	Tree shrew			3–10, 11–20, 21–30 months	DG	#BrdU cells: 3–10 m > 11–20 m > 21–30 m
(Tanaka *etal*., 2007)	Mouse	ICR	Males	2 and 9 months	DG	#Ki67 cells: 2 m > 9 m
					SVZ	#Ki67 cells: 2 m > 9 m
(Tropepe *etal*., 1997)	Mouse	SW/COBS	Males	2–4 and 23–25 months	SVZ	#BrdU cells: 2–4 m > 23–25 m

Despite a drastic drop in proliferation, the short-term ***survival*** pattern of the newborn cells, that is, the proportion of cells that do not die shortly after their birth, seems to be unaffected by aging (see Table 2). A time course study comparing juvenile (38 days) and middle-aged rats (12 months old) has shown a significant peak in the number of BrdU-labeled cells observed 7 days after the injection, independent of the animal's age (McDonald & Wojtowicz, 2005). Following this peak, the number of BrdU-labeled cells rapidly starts to decrease with time. By comparing the ratio between the number of BrdU-positive cells just after BrdU injection and the number of BrdU-labeled cells surviving a few weeks after the injection, several studies have revealed that there is no effect of age on the rate of survival of newborn progeny (Kempermann *etal*., 1998b; Lichtenwalner *etal*., 2001; Bondolfi *etal*., 2004; McDonald & Wojtowicz, 2005; Rao *etal*., 2006). Even 5 months after their birth, the survival of adult-born neurons is independent of the age of the animals (Rao *etal*., 2005).

**Table 2:** Influence of aging on the newborn cell survival and differentiation within the subventricular zone (SVZ) and the dentate gyrus (DG)

Reference	Species	Strains	Sex	Ages	Delay	Area	Results
***Survival of newly generated cells***							
(Bizon *etal*., 2004)	Rat	Long-Evans	Male	7 and 25 months	3 weeks	DG	#BrdU cells:7 m > 25 m
(Bondolfi *etal*., 2004)	Mouse	C57BL/6	Male	2, 12, 18, 24 months	4 weeks	DG	#BrdU cells: 2 m > 12 m > 18 m = 24 mSurvival rate: 2 m = 12 m = 18 m = 24 m
(Cuppini *etal*., 2006)	Rat	SD	Male	2, 5, 12 months	15 days	DG	#BrdU cells: 2 m > 5 m > 12 m
(Jin *etal*., 2003a)	Mouse	CD1	Male	3 and 20 months	1 week	DG	#BrdU cells: 3 m > 20 m#BrdU cells: 3 m > 20 m
(Heine *etal*., 2004a)	Rat	Wistar	Male	6 weeks, 12, 24 months	4 weeks	DG	#BrdU cells: 6w > 12 m = 24 m
(Kempermann *etal*., 1998b)	Mouse	C57BL/6	Female	6 and 18 months	4 weeks	DG	#BrdU cells: 6 m > 18 mSurvival rate: 6 m = 18 m
(Kuhn *etal*., 1996)	Rat	F344	Female	6, 12, 27 months	4 weeks	DG	#BrdU cells: 6 m > 12 m = 27 m
(Lichtenwalner *etal*., 2001)	Rat	BNxF344	Male	5, 18, 28 months	4 weeks	DG	#BrdU cells: 5 m > 18 m = 28 m
(McDonald & Wojtowicz, 2005)	Rat	SD	Male	1 and 12 months	60 days	DG	#BrdU cells: 1 m > 12 mSurvival rate: 1 m = 12 m
(Merrill *etal*., 2003)	Rat	F344	Female	2 and 21 months	10 days	DG	#BrdU cells: 2 m > 21 m
(Rao *etal*., 2005)	Rat	F344	Male	4, 12, 24 months	5 months	DG	Survival rate: 4 m = 10 m = 24 m
(Rao *etal*., 2006)	Rat	F344	Male	4, 12, 24 months	10 days		#BrdU cells: 4 m > 12 m = 24 m
(Segovia *etal*., 2006)	Rat	Wistar	Male	2 and 25 months	6 weeks	DG	#BrdU cells: 2 > 25 m
(Tropepe *etal*., 1997)	Mouse	SW/COBS	Male	2–4 & 23–25 months	31 days	OB	#BrdU cells: 2–4 m > 23–25 m
(van Praag *etal*., 2005)	Mouse	C57BL/6	Male	3 and 19 months	35 days	DG	#BrdU cells: 3 m > 19 m
(Wati *etal*., 2006)	Rat	SD	Male	3–4 and 28 months	1 week	DG	#BrdU cells: 3–4 m > 28 m
***Phenotype of newly generated cells***							
(Bizon *etal*., 2004)	Rat	Long-Evans	Male	7 and 25 months	3 weeks	DG	%BrdU/NeuN: 83 > 67%BrdU/GFAP: 0/5
(Bondolfi *etal*., 2004)	Mouse	C57BL/6	Male	2, 12, 18, 24 months	4 weeks	DG	%BrdU/NeuN: 68 > 39 = 33 = 30%BrdU/S100: 9/15/21/20
(Driscoll *etal*., 2006)	Rat	FBNF1	Female	3, 12, 24 months	5 weeks	DG	%BrdU/NeuN cells: 3 m > 12 m > 24 m
(Enwere *etal*., 2004)	Mouse	C57BL/6	Male	2 and 24 months	4 weeks	OB	%BrdU/TH: 2 m > 24 m%BrdU/calretin: 2 m > 24 m
(Heine *etal*., 2004a)	Rat	Wistar	Male	12 and 24 months	4 weeks	DG	%BrdU/NeuN: 12 m > 24 m
(Kempermann *etal*., 1998b)	Mouse	C57BL/6	Female	6 and 18 months	4 weeks	DG	%BrdU/NeuN: 6 m > 18 m%BrdU/S100 : 6 m < 18 m
(Lichtenwalner *etal*., 2001)	Rat	BNxF344	Male	5, 18, 28 months	4 weeks	DG	%BrdU/NeuN: 5 m > 18 m = 28 m
(McDonald & Wojtowicz, 2005)	Rat	SD	Male	1 and 12 months	3, 7, 8, 14, 21, 60 days	DG	%BrdU/DCX: 1 m > 12 m%BrdU/CaBP: 1 m > 12 m
(Molofsky *etal*., 2006)	Mouse	C57BL/6	Male	2 and 24 months	4 weeks	OB	%of NeuN/BrdU: 2 m > 24 m
(Nacher *etal*., 2003)	Rat	F344	Female	3, 10, 20 months	3 weeks	DG	%BrdU/rCRMP4: 3 m > 10 m > 20 m
(Rao *etal*., 2005)	Rats	F344	Male	4, 12, 24 months	10 days5 months	DG	%BrdU/DCX: 4 m = 12 m = 24 m%BrdU/DCX: 4 m = 12 m = 24 m%BrdU/NeuN: 4 m = 12 m = 24 m
(van Praag *etal*., 2005)	Mouse	C57BL/6	Male	3 and 19 months	35 days	DG	%BrdU/NeuN: 3 > 19 m%BrdU/S100b: 3 m < 19 m

The column ‘Delay’ refers to the interval between the labeling of newly generated cells by BrdU injections and the sacrifice of the animals.

In addition to changes in proliferation, the capacity of the newly born cells to ***migrate*** radially from their birthplace in the SGL toward the GCL also slows down with increasing age (Heine *etal*., 2004a; Rao *etal*., 2005). In fact, in aged animals, only a small fraction of the newly born cells is able to migrate to the GCL within 4 weeks (Heine *etal*., 2004a). However, 5 months after their birth, the position of newly born cells within the GCL of aged subjects is similar to that of their young counterparts (Rao *etal*., 2005). This delayed migration may be because of an age-related decline in the expression of a highly polysialylated neural cell adhesion molecule, which is associated with the migration and maturation of immature precursor cells (Seki & Arai, 1995; Ni Dhuill *etal*., 1999).

The ***phenotype*** of 2-week-old to 6-week-old newborn cells is determined by double labeling of BrdU-labeled cells with immature neuronal markers (e.g. doublecortin), mature neuronal markers (e.g. calbindin or neuron-specific nuclear protein, NeuN), or glial markers (e.g. GFAP, see Table 2). Most studies have shown a strong reduction in the differentiation into neuronal phenotypes in aging subjects (Kempermann *etal*., 1998b; Lichtenwalner *etal*., 2001; Nacher *etal*., 2003; Bizon *etal*., 2004; Bondolfi *etal*., 2004; Heine *etal*., 2004a; McDonald & Wojtowicz, 2005; Driscoll *etal*., 2006; Molofsky *etal*., 2006). In the most drastic case reported, almost 70% of newly born cells differentiated into neurons (Bizon *etal*., 2004). However, some groups were not able to reproduce these results and have reported only small (McDonald & Wojtowicz, 2005) or undetectable changes in the proportion of new cells differentiating into neurons (Rao *etal*., 2006). These discrepancies are likely because of the fact that either the animals were significantly younger or the survival period between BrdU injection and animal perfusion was different. In some studies, an increase in astrocytic differentiation was also observed in aged animals (Bizon *etal*., 2004; Bondolfi *etal*., 2004).

Additionally, analysis of dendritic growth in 12-day-old BrdU–DCX double-labeled neurons revealed that dendritic maturation was considerably reduced during aging. At that time, newly born neurons exhibited diminished dendritic branching and total dendritic length compared with their age-matched counterparts in young DG (Rao *etal*., 2006). However, another study labeling adult-born cells with a GFP retrovirus found that dendritic length, branching, and spine density of 4-week-old cells were similar in young and aged brain (van Praag *etal*., 2005). The discrepancy between these two studies could be related to the age of the cells studied (2 weeks vs. 4 weeks). Another possibility is that in one study (van Praag *etal*., 2005), dendritic analysis was performed after running, a condition that might accelerate the maturation of adult-born neurons. Clearly, some additional experiments are needed to better understand the effect of aging on dendritic maturation.

### In the SVZ

Within the SVZ, the existence of age-related changes in cell proliferation is not so clear and may show important variations among species (see Tables 1 and 2). In mice, a two- to threefold reduction of cell proliferation in the SVZ has been reported using BrdU labeling in young adults (2–5 months) compared to aged (20–27 months) mice (Tropepe *etal*., 1997; Jin *etal*., 2003a; Enwere *etal*., 2004; Maslov *etal*., 2004; Molofsky *etal*., 2006). In rats, however, no difference in the density of BrdU-labeled cells was observed between 6-month-old and 21-month-old rats (Kuhn *etal*., 1996). Aside from possible species-specific differences, this discrepancy can likely be explained by the fact that in the SVZ, newborn cells quickly leave the proliferation area to migrate toward the OB. Consequently, slight differences in the BrdU injection protocol may strongly affect the labeling index of SVZ cells and make the differences between young and aged individuals undetectable. Indeed, the use of intrinsic markers of cell proliferation expressed by proliferating and/or relatively quiescent cells such as MCM-2, Ki67, and HH3 showed a substantial reduction of neurogenesis in the SVZ of aged rats compared with young adult rats (Zhang *etal*., 2006; Tanaka *etal*., 2007). Electron microscopy studies have revealed that the proliferation defect is specific to neuroblasts and transitory amplifying progenitor cells, which are restricted to the anterior dorsolateral horn of the SVZ, while the number of SVZ stem cells remains relatively constant (Luo *etal*., 2006). Moreover, *in vitro* experiments suggest that an age-related decrease occurs in the number of restricted progenitors, but not in the number of stem cells (Tropepe *etal*., 1997), thus indicating that SVZ progenitors and stem cell numbers are differentially regulated with age. In agreement with this, the number of BrdU–GABA double-labeled neurons in the granule and glomerular layers of the OB was halved in aged mice (Enwere *etal*., 2004). This was accompanied by a reduction of BrdU-labeled periglomerular neurons expressing tyrosine hydroxylase (TH, 71%) or calretinin (59%).

### Conclusions

Cell proliferation is the stage of neurogenesis in the DG and the SVZ that is most affected by aging. In contrast, migration, survival, and neuronal fate choice seem to be less dramatically affected, as they seem only to be delayed in the aged DG. Given the ‘paucity’ of data in the SVZ, it is unknown whether aging affects migration (tangentially along the RMS or radially within the OB), cell survival, or cell differentiation. Remarkably, after a pronounced increase in the first few postnatal weeks, the volume of the GCL and the total number of granule cells in the DG appear to be essentially stable throughout life (West, 1993; West *etal*., 1994; Rapp & Gallagher, 1996; Rasmussen *etal*., 1996; Kempermann *etal*., 1998b; Merrill *etal*., 2001; Rapp *etal*., 2002; Jin *etal*., 2003a; Heine *etal*., 2004a), suggesting that aging affects the turnover of granule cells rather than their absolute number. This hypothesis is strengthened by the fact that apoptosis slows down profoundly in the DG (Heine *etal*., 2004a). However, it is obvious that further studies are needed to: (i) distinguish the effects of aging on the activity of slowly cycling stem or stem-like cells and the more rapidly cycling transit progenitors; (ii) better characterize the effects of aging on the development of new cells in an old environment; and (iii) study the electrophysiological properties of these neurons.

## What are the mechanisms involved in the decrease of neurogenesis?

Age-related changes in neurogenesis could be the consequence of the inability of old precursors to respond appropriately to external stimuli because of changes in their intrinsic properties. Alternatively, age-related changes that occur in local and systemic environments might be responsible for the decline in neurogenesis.

### Old cells?

Overall, the data available from the bibliography suggest that the main difference between juvenile/young and aged rats is a decrease in the rate of cell proliferation. However, the mechanisms responsible for this effect are still unclear. Lengthening of the cell cycle occurring throughout the lifelong proliferative period could be responsible for the reduction in the rate of neuronal production that occurs with age. Alternatively, decreased neurogenesis could result from a progressive loss of precursors. Finally, those precursors might become quiescent with age, even though they could still retain the potential for reactivation. Ultimately, the age-related decline in neurogenesis may be the consequence of not just one of these phenomena but, more likely, a combination of them.

#### In the DG

As there are no specific markers for stem/progenitor cells in the adult brain, it is difficult to determine their precise number throughout life. Cumulative BrdU labeling, as well as endogenous markers of cell proliferation, suggest that the size of the dividing precursor pool is three to four times smaller in middle-aged animals compared to young adult animals (Olariu *etal*., 2007). GFAP-, nestin-, and vimentin-expressing radial-like cells are believed to be the precursor cells that divide asymmetrically to generate a daughter radial cell and a DCX-expressing direct progenitor (Seri *etal*., 2004; Encinas *etal*., 2006). Similar to what is observed with cumulative labeling studies, the number of these cells in the GCL is also dramatically decreased with age, becoming very low in both middle-aged and aged animals (Alonso, 2001; Nacher *etal*., 2003). However, these approaches do not take into consideration the existence of quiescent precursors. Using Sox-2 as a putative marker of stem cells, it has been shown that the overall number of Sox-2 cells remains constant in young, middle-aged, and aged rats, yet the percentage of these cells expressing proliferation markers (BrdU and Ki67) is drastically reduced with age (Hattiangady & Shetty, 2008). These results suggest that aging is not associated with a decrease in the total number of precursors in the SGL, but, rather, with a decrease in the proportion of active precursors, as a result of increased quiescence of these cells, likely because of age-related changes in the surrounding milieu. Moreover, a comparison of Sox-2/BrdU data with Sox-2/Ki67 results suggests a lengthening of the cell cycle of NSCs between young adult and middle-aged F344 rats as well as between middle-aged and aged F344 rats (Hattiangady & Shetty, 2008). However, this result was not confirmed by a study carried out on young adult and 10-month-old Sprague–Dawley rats, using a protocol of multiple BrdU and tritiated thymidine injections coupled with endogenous proliferation markers, specifically designed to measure the size of the proliferating population and its cell cycle duration (Olariu *etal*., 2007). Additional studies on older animals are now required to definitively corroborate or rule out the possibility of changes in the cell cycle with advancing age.

#### In the SVZ

Only a few studies have examined the mechanisms involved in the decrease of neurogenesis in the aged SVZ, leading to contradictory results. On one hand, sequential labeling with BrdU and tritiated thymidine has revealed a lengthening of the cell cycle in the SVZ of older mice (Tropepe *etal*., 1997; Jin *etal*., 2003a). On the other hand, the use of a combination of proliferation markers specific for the different stages of the cell cycle to identify the fraction of the proliferative progenitors that are in S-phase at a given time has demonstrated that aging does not change the rate of division of SVZ cells (Maslov *etal*., 2004). However, consistent with an increase in the length of the cell cycle, the time course for repopulation after a complete depletion of constitutively proliferating cells in the SVZ by an AraC treatment (see Doetsch *etal*., 1999b for protocol) is markedly different in aged animals compared to their younger counterparts. Complete recovery was not seen until 14 days in aged animals compared to only 4–8 days in young adult animals (Enwere *etal*., 2004). These experiments suggest that the reduction in proliferation detected in the SVZ might result from a lengthening of the cell cycle rather than from a reduction in the number of precursors. However, it is unknown if this lengthening is an intrinsic property of the aging SVZ stem/progenitor cells or if it is the consequence of changes in the environment, for example, the influence of inhibitory factors and/or absence of a stimulatory factor.

#### Conclusion

When taken together, these results suggest that the drastic decline of neurogenesis observed during aging may be attributable to several mechanisms (Fig. 2), for example, a decreased number of proliferating cells or limited proliferation potential resulting from increased quiescence.

### Old environment?

In this context, either newly born cells may have lost their intrinsic capacity to respond to the mitotic stimuli provided by the environment (e.g. growth factor receptors) or, alternatively, the local environment may have changed so that the mitotic stimuli are no longer provided (see Table 3). The rapid reduction in the rate of proliferation and migration during aging is intriguing, as it suggests that the fate of newborn cells is strongly influenced by local environmental factors rather than by intrinsic or genetic cues. This hypothesis is strongly supported by the fact that: (i) during aging, positive regulators of neurogenesis are known to decrease, whereas signals identified as neurogenesis inhibitors are increased; and (ii) neurogenesis in the aging brain can be boosted by increasing the level of pro-neurogenic factors or by decreasing the levels of anti-neurogenic factors (see below). It has been suggested that this effect does not result from accelerating the cell cycle of the precursors, but from an increase in the number of precursors in aged DG (Olariu *etal*., 2007). The additional dividing cells could potentially come from precursors that have become quiescent over time, that is, cells that are not dividing but have retained their capacity to divide. Consequently, in the senescent brain, the adult neural precursor cells can apparently stop dividing for long periods of time, but still proliferate when prompted by the right signal.

#### Steroids

##### Corticosteroids.

Corticosteroids have been identified as one of the factors having the strongest negative regulatory effects on adult hippocampal neurogenesis. Corticosteroids are released into the blood circulation following the activation of the hypothalamo–pituitary–adrenal (HPA) axis, primarily by stress. Corticosterone, the main corticosteroid in rodents, regulates its own secretion through negative feedback by interacting with two receptors present in the DG, the mineralocorticoid receptor (MR) and the glucocorticoid receptor (GR) (van Eekelen *etal*., 1991; Sapolsky *etal*., 2000). Mineralocorticoid receptors have a higher affinity for corticosterone than GRs, and are primarily found in limbic structures, including the HF (van Eekelen *etal*., 1988), whereas GRs are expressed ubiquitously. Acute (Cameron & Gould, 1994) or chronic (Ambrogini *etal*., 2002) treatment with corticosterone has been associated with a strong down-regulation of cell proliferation in the adult DG. Different paradigms of stress, which dramatically increase corticosterone levels, decrease either cell proliferation (Gould *etal*., 1997, 1998; Czeh *etal*., 2001; Pham *etal*., 2003; Heine *etal*., 2004b; Simon *etal*., 2005) or cell survival and neuronal differentiation in the DG (Pham *etal*., 2003; Thomas *etal*., 2007). This effect is reversible, as 3 weeks after the termination of the stress stimulus, a total recovery is observed (Heine *etal*., 2004b). On the other hand, short-term suppression of corticosterone secretion by adrenalectomy in adulthood increases granule cell neurogenesis (Gould *etal*., 1992; Cameron & Gould, 1994). This change can be prevented by corticosterone replacement or by stimulation of MR or GR (Gould *etal*., 1992; Rodriguez *etal*., 1998; Montaron *etal*., 2003). In the young DG, GRs are expressed by 50% of the stem-like astrocytes, early progenitors, and immature new neurons, whereas MRs seem to be expressed only by more mature, calbindin-positive granule cells (Garcia *etal*., 2004a). However, the presence of MRs in mature neurons is inconsistent with the fact that the stimulation of these receptors is sufficient to reverse the adrenalectomy-induced increase in cell proliferation (Montaron *etal*., 2003; Wong & Herbert, 2005).

The age-related decline in neurogenesis has been associated with increased exposure to corticosterone, resulting from increased basal levels, mainly during the dark phase of the circadian cycle, and prolonged stress-induced secretion (Meaney *etal*., 1992; Sapolsky, 1992; Lupien *etal*., 1994). This intriguing correlation has led to the hypothesis that chronically elevated corticosterone levels are responsible for reduced neurogenesis in the aging DG. Consequently, the effects of adrenalectomy in senescent rats were studied. Adrenalectomy in senescent rats dramatically reduces corticosterone levels (below 0.3 µg dL^−1^) (Montaron *etal*., 1999), and 1 week later, increases cell proliferation and, consequently, neurogenesis (Cameron & McKay, 1999; Montaron *etal*., 1999). This effect depends on corticosterone secretion because it can be prevented by treatment with the hormone (Montaron *etal*., 1999). This set of experiments was the first to indicate that the age-related decrease in neurogenesis may not be solely caused by a limitation of the stem cells themselves, but rather, to inadequate environmental signals. Adrenalectomy at midlife blocked the age-related increase in both basal- and stress-induced corticosterone secretion and, subsequently, increased cell proliferation and neurogenesis in senescent animals (Montaron *etal*., 2006). When adrenalectomy is performed during early postnatal life and treatment with corticosterone is given orally, the rate of neurogenesis in middle-aged rats does not differ from that of age-matched, sham-operated controls (Brunson *etal*., 2005). However, differences in corticosterone levels [10 µg dL^−1^ in middle-aged, adrenalectomized rats (Brunson *etal*., 2005) compared to less than ≤ 2 µg dL^−1^ in 3-month-old rats and to aged rats adrenalectomized at either middle age or senescence (Montaron *etal*., 1999, 2006)] might explain this discrepancy. Using an approach that takes into account the individual differences in the activity of the HPA axis, we have shown that in aged animals, the weight of the adrenal glands, considered as a reliable index of the chronic activity of the HPA axis, is inversely correlated with the proliferation and survival of BrdU-labeled cells in the GCL (Montaron *etal*., 2006). In other words, hyperactivity of the HPA axis is associated with a low level of cell proliferation and survival. When compared to young animals, aged rats have higher expression of GR in early precursors, and calretinin-positive immature neurons express both GR and MR (Garcia *etal*., 2004a). This shift of the GR and MR expression profile toward a more immature stage of neuronal development suggests increased steroid sensitivity of the aged DG to corticoid impregnation.

In conclusion, long-term exposure to high levels of corticosterone throughout the animal's life can damage the precursor cell population, permanently decreasing the size of the dividing population in aged animals. Alternatively, corticosterone may be directly affecting proliferation without damaging the precursors, resulting in a reversible decline in neurogenesis.

##### Neurosteroids.

Neurosteroids are a subclass of steroids synthesized *de novo* in the brain, in particular, in the HF (Baulieu & Robel, 1997). In young adult rats, intracerebroventricular (icv) infusion of allopregnanolone, a neurosteroid which acts as a positive allosteric modulator of the GABA_A_ receptors that are present on neuroblasts, decreases hippocampal cell genesis. In contrast, other neurosteroids such as pregnenolone sulfate (Preg-S) and dehydroxyepiandrosterone acting as negative allosteric modulators of GABA_A_, increase hippocampal neurogenesis (Karishma & Herbert, 2002; Mayo *etal*., 2003). The effects of Preg-S on neurogenesis are probably mediated by GABA_A_ receptors because the Preg-S-induced increase in neurogenesis can be blocked by prior icv administration of muscimol, a GABA_A_ agonist. Pregnenolone sulfate may act directly on these receptors because it is able to stimulate the proliferation of neural spheres *in vitro* (Mayo *etal*., 2003). In aged animals, acute icv infusion of Preg-S considerably increases the rate of cell proliferation and neurogenesis (Mayo *etal*., 2003). Interestingly, it has been proposed that age-related alterations are caused by an abnormally strong inhibitory GABAergic input (Marczynski, 1998; Segovia *etal*., 2006). Together with the observation that Preg-S increases neurogenesis, this suggests that the age-related decline in neurogenesis could be related to an enhancement of GABA transmission.

#### Glutamate

Granule cells are glutamatergic in nature and receive glutamatergic afferents mainly from the entorhinal cortex through the perforant pathway (Vizi & Kiss, 1998). In the young adult DG, the blockade of NMDA receptors by competitive or noncompetitive receptor antagonists enhances the number of newly generated granule neurons. This suggests an inhibitory action of glutamate on neurogenesis (Cameron *etal*., 1995; Nacher *etal*., 2001). Moreover, NMDA receptors and corticosterone are believed to work synergistically in inhibiting cell proliferation (Cameron *etal*., 1998). In aged rats, neurogenesis can be reactivated by intraperitoneally injecting NMDA receptor antagonists, which elicit a significant increase in the number of stem-like cells, proliferating cells, and new neurons in the DG (Nacher *etal*., 2003).

#### Growth factors

##### Epidermal growth factor (EGF) signaling.

Among the various members of the EGF family of ligands that are able to interact with EGF receptor (EGFR), only three have been shown to be expressed in the brain: (i) transforming growth factor alpha (TGFα), which binds only the EGF-R and is supposed to be the main endogenous ligand in the brain (Wilcox & Derynck, 1988; Seroogy *et al*., 1991, 1993; Lazar & Blum, 1992); (ii) heparin-binding EGF-like growth factor (HB-EGF), also able to bind a related receptor, ErbB4; and (iii) amphiregulin, to a lesser extent (Opanashuk *et al*., 1999). In the adult SVZ, icv administration of EGF expands the precursor population. This is accompanied by a differentiation bias toward the astrocyte phenotype, ultimately leading to a reduction in the total number of newborn neurons that reach the OB (Kuhn *et al*., 1997). Heparin-binding EGF-like growth factor seems to have a different effect as it is able to increase both cell proliferation in the SVZ and the number of newly born neurons reaching the OB (Jin *et al*., 2002a, 2003b). In the DG, HB-EGF (Jin *et al*., 2002a, 2003a) but not EGF (Kuhn *et al*., 1997) increases cell proliferation and neuronal differentiation (Jin *et al*., 2002a), while the role of TGFα is still unknown.

During aging (between 2 months and 24 months in mice), both EGFR and TGFα expression (at least in the SVZ) decline by 50% and 70%, respectively (Enwere *etal*., 2004). This suggests that the expansion potential of NSC progeny may be reduced because of a reduction in EGFR signaling. The consequences of HB-EGF infusion in the aged brain are similar, yet more significant, than the effects observed in young adults. Indeed, HB-EGF increases cell proliferation by ~1.6- and 5.5-fold in young and aged DG, respectively, and ~2.4- and ~2.7-fold in the young and aged SVZ, respectively. In the end, the number of BrdU-labeled cells is comparable in untreated 3-month-old and treated 20-month-old mice (Jin *etal*., 2003a). On the other hand, EGF is less efficient at increasing cell proliferation in the aged SVZ when compared to HB-EGF (~1.8-fold increase in young adult and ~1.4-fold in aged animals) (Enwere *etal*., 2004). The differences observed between these EGFR ligands are likely because of the different receptors and intracellular pathways involved.

##### Insulin-like growth factor-I (IGF-I).

Among the growth factors that may regulate neurogenesis, IGF-I is of particular interest given its expression pattern throughout life. Insulin-like growth factor-I is strongly expressed during development (Bondy, 1991; Baker *et al*., 1993), but its expression is subsequently gradually reduced in the adult brain. However, it persists in adult neurogenic areas (Rotwein *et al*., 1988; Anlar *et al*., 1999), most likely because of its local production by glial cells (Fernandez-Galaz *et al*., 1997; Du & Dreyfus, 2002). Insulin-like growth factor-I has considerable influence in adult hippocampal neurogenesis, as it stimulates both cell proliferation and neuronal differentiation (Aberg *et al*., 2000; Trejo *et al*., 2001; Anderson *et al*., 2002). During aging, IGF-I and IGF-I receptor levels undergo a secondary decline (Sonntag *et al*., 1999; Lai *et al*., 2000; Shetty *et al*., 2005), especially in the HF. Consequently, the reduced IGF-I concentration observed from middle age may contribute to initiating the age-related decline in neurogenesis. Indeed, the decrease of IGF-I levels with age correlates with the evolution of neurogenesis and can be reversed by icv infusion of IGF-I (Lichtenwalner *et al*., 2001). This treatment triples the number of newborn neurons in aged rats through a remarkable increase in cell proliferation.

##### Fibroblast growth factor 2 (FGF-2).

Fibroblast growth factors (FGFs), in particular FGF-2 (also called basic FGF), have been shown to play an important role during CNS development by controlling neurogenesis, neuron survival, and differentiation (Walicke *etal*., 1986; Morrison *etal*., 1988; Vicario-Abejon *etal*., 1995; Nakagami *etal*., 1997; Vaccarino *etal*., 1999). The expression of FGFs and FGF receptors (FGFRs) persists in the adult brain. Strikingly, the astrocytes of the DG, the SVZ, the RMS, and the OB display the most robust FGF receptor 2 (FGFR-2) expression in the adult brain (Chadashvili & Peterson, 2006). This result points out the possible role of FGF signaling in neurogenesis. In adult SVZ, a proliferative effect of FGF-2 as well as an enhancement of migration to the OB has been clearly demonstrated (Kuhn *etal*., 1997; Wagner *etal*., 1999; Jin *etal*., 2003a). In the adult DG, similar experiments increase proliferation in the hilus but are ineffective in the SGL (Kuhn *etal*., 1997; Jin *etal.* 2003a). On the other hand, over-expression of FGF-2 by gene transfer in lifelong FGF-2-deficient mice up-regulates DG cell proliferation (Yoshimura *etal*., 2001).

The hippocampal concentration of FGF-2 decreases between young adult age and middle age, but shows no change between middle age and old age. This decrease is more particularly associated with the decline of a subpopulation of astrocytes that express FGF-2 and could be the consequence of an age-related impairment in FGF-2 synthesis by astrocytes (Shetty *etal*., 2005). In this manner, an age-related decrease in FGFR-2 levels is observed in the SVZ, RMS, OB, and HF, but not in non-neurogenic regions of the brain (Chadashvili & Peterson, 2006). Unlike what is observed in young adults, FGF-2 icv infusion for 2 weeks or 3 days increases neurogenesis in both the DG and the SVZ of middle-aged (Rai *etal*., 2007) and aged mice (Jin *etal*., 2003a). The enhanced production of new neurons was associated with an enhanced dendritic growth (Rai *etal*., 2007). Thus, age-related declines in hippocampal neurogenesis are likely linked to reduced FGF-2 concentrations.

##### Vasculature and vascular endothelial growth factor (VEGF).

The vasculature is an important component of adult neurogenic niches. Blood vessels are conduits for the delivery of long-distance paracrine factors (e.g. hormones, growth factors.) from distant sources, and by this means, they could play an essential indirect role in the regulation of neurogenesis. Furthermore, in the DG (Palmer *etal*., 2000; Heine *etal*., 2005) as well as in the SVZ (Bovetti *etal*., 2007), new cells are clustered in close proximity to blood vessels where VEGF expression is high and angiogenesis is ongoing (Palmer *etal*., 2000). It is, thus, believed that neurogenesis and angiogenesis are mechanistically linked, and that VEGF, which is normally expressed in cerebral microvessels, is the linking factor between these two events (Palmer *etal*., 2000; Jin *etal*., 2002b). Consistent with this hypothesis, several studies performed in young adult rodents have highlighted the importance of VEGF in adult bulbar and hippocampal neurogenesis (Jin *etal*., 2002b; Fabel *etal*., 2003; Sun *etal*., 2003; Cao *etal*., 2004; Greenberg & Jin, 2004).

Brain aging is associated with a reduction in the cerebral microvasculature (Sonntag *etal*., 1997), a loss of microvascular plasticity (Riddle *etal*., 2003), and reduced VEGF synthesis (Shetty *etal*., 2005). This is specifically true in the DG where the total volume of the SGL occupied by RECA-1^+^ capillaries undergoes a more than 25% decrease in aged compared to young adult animals (Hattiangady & Shetty, 2008). Thus, limited angiogenesis, decreased cerebral blood flow, and decreased concentration of the associated growth factor, VEGF, in the aged brain may contribute to the decline in cell genesis. Using Sox2 as a marker of neural stem/progenitor cells, it has been shown that the distance between endothelial cells and the putative stem cells is increased with aging. This may in turn reduce the accessibility of these cells to endothelial-cell-derived and blood-transported factors (Hattiangady & Shetty, 2008).

#### Cell cycle regulators

The polycomb transcriptional repressor Bmi-1 is required for the self-renewal and postnatal maintenance of hematopoietic (Lessard & Sauvageau, 2003; Park *etal*., 2003) and neural stem cells (Molofsky *etal*., 2003, 2005). The absence of Bmi-1 in Bmi-1-deficient mice induces a premature senescence of stem cells and, consequently, a severe reduction in the rate of proliferation in the SVZ, both *in vitro* and *in vivo*. Conversely, Bmi-1 over-expression can prevent senescence and extend the replicative lifespan of primary cells (Molofsky *etal*., 2005). Bmi-1 acts through the repression of two inhibitors of cell proliferation whose induction has also been associated with cellular senescence: p16^Ink4a^, a cyclin-dependent kinase inhibitor, and p19^Arf^, which promotes p53 activation (Jacobs *etal*., 1999; Sherr, 2001). Indeed, deletion of Ink4a or Arf from Bmi-1^−/–^ mice partially rescued stem cell self-renewal and stem cell proliferation defects in the SVZ (Bruggeman *etal*., 2005; Molofsky *etal*., 2005).

p16^INK4a^ Gene expression increases with age in a variety of tissues (Zindy *etal*., 1997; Krishnamurthy *etal*., 2004), including the SVZ where p16^INK4a^ expression is not detectable in the SVZ of 60-day-old mice, but becomes detectable by 1 year of age and is further increased at 2 years of age (Molofsky *etal*., 2006). On the other hand, p19^Arf^ expression in the SVZ is not affected by aging. The effects of p16^INK4a^ on the generation of new neurons in the SVZ increase with age and have been studied employing p16^INK4a^-deficient mice. While no effect was observed in young adults, p16^INK4a^ deficiency significantly increases the frequency of newly generated OB neurons in aged animals. Notably, p16^INK4a^ deficiency does not affect the ratio of non-neuronal cells in the OB or neurogenesis in the DG (Molofsky *etal*., 2006). Thus, in certain regions such as the SVZ but not the DG, stem cell function is regulated by a balance between Bmi-1, which promotes stem cell maintenance and regenerative capacity, and tumor suppressors like p16^INK4a^, which reduce regenerative capacity and promote aging. During aging, this balance is probably affected by as yet unidentified factors resulting in a reduction of precursor function and neurogenesis in at least certain regions of the nervous system.

#### Conclusion

The studies conducted so far have clearly showed that corticosteroids exert a deleterious influence on hippocampal neurogenesis during aging (see also the third section). Although the list of factors influencing the course of neurogenesis is growing, little is known about their influence in the aging brain. This is because not only of the inherent difficulty of *in vivo* aging studies, but also to the controversy over the effects of some factors cited earlier in young rats, for example, glutamate and GABA have also been shown to promote neurogenesis (Deisseroth *etal*., 2004; Ge *etal*., 2007). Furthermore, *in vivo*‘pharmacological’ studies involve changes in local networks (other neurons and other types of cells in the DG), in the structure (HF) and in connected structures. Thus, the observed effects are certainly not only the result of a direct action on the precursors or their lineage.

The changes observed during senescence cannot be explained by only one of the listed factors, and are a consequence of intricate regulation of different factors working in concert and often dependent on each other. They can play either permissive or instructive roles, and it is likely that there is a strictly orchestrated regulation of all of them, where some could be involved at early stages of aging and others could take effect only at later times, leading to the aging of neurogenic areas. This environmental-dependent regulation of neurogenesis supports the idea that the age-related loss of new neurons is not an irreversible cell-intrinsic process and shows that, when triggered by appropriate signals, neurogenesis can be reactivated in senescent brain.

A reduction of neuronal plasticity is hypothesized to be the cornerstone of the appearance of age-related deficits. The possibility of preventing it by manipulating the basal level of neurogenesis raises new hope of improving brain function during aging. Consequently, in the last part of this review, we will examine the known relationships between the age-related decline in neurogenesis and age-dependent memory impairment, and see to what extent the low rate of neurogenesis can contribute to the appearance of deficits.

## Functional consequences of the age-related decrease in neurogenesis

Several lines of evidence based on the structure–function relationships support the involvement of neurogenesis in memory processing in the young adult brain. In particular, adult-born olfactory neurons have been shown to be involved in olfactory memory, while adult-born hippocampal neurons have been related to complex forms of spatial or associative memories (Aimone *etal*., 2006; Leuner *etal*., 2006; Lledo *etal*., 2006; Abrous & Wojtowicz, 2008). The fact that adult neurogenesis strongly decreases with age raises the important question of whether this decline in plasticity participates in the appearance of age-related dysfunction and whether a reduced number of new cells in an old brain can make a relevant functional contribution. Apart from an elegant study by Enwere *etal*. (2004) showing that the age-related decline in adult-born periglomerular neurons is associated with deficits in fine odor discrimination, most studies on aging have focused on the role of adult hippocampal neurogenesis in hippocampal functioning and, especially, in spatial memory. The spatial learning deficits observed in senescent animals are similar to those caused by hippocampal alterations (Rosenzweig & Bennett, 1996; Morris, 2006); this has raised the critical issue as to whether an alteration of hippocampal neurogenesis could be responsible for the age-related loss of spatial memory abilities. From an operational standpoint, the water maze has been the only test used, with the exception of one study. This paradigm requires that animals learn multiple extra-maze visual cues, allowing them to build a dynamic spatial representation of their surroundings for navigating to a platform hidden underneath the surface of the water. The requirement of having to learn complex relationships of extra-maze visual cues is the aspect of the test that renders it sensitive to hippocampal dysfunction.

In the following sections, we will review the evidence linking neurogenesis and memory during ‘normal’ aging. By this term, we refer to the natural process of memory decline that does not involve neurodegenerative processes such as those observed in Alzheimer's disease. A distinction will be made between successful and pathological aging, the latter – and not the former – being characterized by the appearance of memory deficits.

### Spatial memory abilities and neurogenesis in old rats

It has long been recognized that spatial learning is particularly vulnerable to the effects of aging. However, memory alteration is extremely variable within a population, and not all experimental animals exhibit memory disorders (Gage *etal*., 1988; Markowska *etal*., 1989; Rapp & Amaral, 1992; Gallagher *etal*., 1993). In particular, some old animals show a clear impairment of spatial reference memory using the water maze, while others exhibit memory capacities similar to those of younger individuals (Gage *etal*., 1988; Markowska *etal*., 1989; Rapp & Amaral, 1992; Gallagher *etal*., 1993). The impairments observed in some aged animals are similar to those observed after hippocampal lesions (Redish & Touretzky, 1998; Stoelzel *etal*., 2002) and have been associated with defects in hippocampal circuitry and plasticity (Petit & Ivy, 1988; Markowska *etal*., 1989; Gallagher *etal*., 1990; Rapp & Amaral, 1992; Rowe *etal*., 2007). The hypotheses being pursued relate the ability to perform hippocampus-related functions to hippocampal neurogenesis in basal (off the learning phase) and dynamic (in the course of learning) conditions.

#### The rate of basal neurogenesis determines learning performance and memory

The existence of a correlation between spatial memory ability, cell proliferation, cell survival, and neurogenesis was examined under basal conditions. In these circumstances, spatial memory performance of aged rats was found to predict the level of hippocampal neurogenesis. Indeed, memory abilities were positively correlated to the number of proliferating cells, surviving cells, and new neurons evaluated 3 weeks *after* training. In other words, animals with preserved spatial memory [aged unimpaired (AU)] exhibited a higher level of proliferating cells, 1-month-old surviving cells, and new neurons compared to animals displaying spatial memory impairments [aged impaired (AI)]. This *quantitative* relationship between a reservoir of new neurons and memory capability – revealed by linking, for a given individual, the levels of memory performances, and the levels of neurogenesis – reinforces the contention that neurogenesis participates in learning and memory. A similar correlation was observed using two different hippocampal-dependent tasks: a modified version of the water maze and a transverse patterning discrimination (visual discriminations) task (Driscoll *etal*., 2006). In these two tasks, performance was shown to be correlated with both hippocampal volume (measured by *in vivo* MRI) and neurogenesis assessed by DCX. However, two other studies failed to demonstrate a correlation between cell proliferation and spatial memory (Bizon & Gallagher, 2003; Merrill *etal*., 2003), and another one reported that greater numbers of 3-week-old BrdU-positive cells were associated with worse memory performance (Bizon *etal*., 2004). Various experimental differences in BrdU injection time (i.e. immediately or 1 week after the completion of the behavioral study as longer intervals are required to make sure that the effects observed are specific to the basal rate of neurogenesis and not a consequence of the recent training; see also below), number of subjects, rat strain, and gender of the animals could explain this apparent controversy.

#### Influence of learning on neurogenesis

Hypothetically, age-related memory deficits could result not only from an alteration of the new neuronal pool, but also from changing the dynamics of hippocampal neurogenesis. To this end, the influence of spatial learning on the birth and/or survival of adult-born cells has been examined. Indeed, in young rats, spatial learning has been reported to influence the production and fate of the newly born cells, depending on the time at which the cells were generated relative to learning. Learning increases the survival of cells born before the beginning of learning (Gould & Tanapat, 1999; Dupret *etal*., 2007), while the survival of cells generated during the early phase of learning (when performance improves rapidly) is decreased by the late phase of learning (when performance is stabilized) (Dobrossy *etal*., 2003). Furthermore, this late phase also shows an increase in cell proliferation (Dobrossy *etal*., 2003). The learning-induced decrease in BrdU cell number was found to reflect the elimination of young neurons by apoptosis (Dupret *etal*., 2007). These three events – survival of relatively mature neurons, apoptosis of more immature cells, and proliferation of precursors – are in fact interrelated events that may mediate learning. Indeed, blocking learning-induced apoptosis inhibits cell survival and cell proliferation, and impairs memory performance (Dupret *etal*., 2007).

In aged rats, spatial learning also influences the fate of the newly born cells according to their birth date and to individual memory abilities (Drapeau *etal*., 2007). In AU rats, learning increases the survival of cells generated at least 1 week before the learning episode, whereas it decreases survival of cells produced during the early phase of learning. In AI rats, cell survival was not influenced by learning. These data indicate that learning, and not training, decreased the survival of adult-born cells, and that spatial memory abilities critically depend on dynamic regulation of adult-born cell survival. Moreover, in contrast to what has been observed in young rats, the late phase of learning (nor the earliest phase) did not increase the proliferation of the cells produced concurrently with this phase. However, given that aging might delay the process leading to neurogenesis, we hypothesized that the learning-induced increase in cell proliferation may also be delayed in the old brain. To this end, cell proliferation was examined 9–14 days after the completion of spatial training. Unexpectedly, cell proliferation was negatively correlated to memory ability: cell proliferation was higher in rats unable to master the task in comparison to animals displaying preserved spatial memory. This surprising result, explained in terms of enhanced swimming activity, is consistent with a previous study (Bizon *etal*., 2004). In this case, the number of surviving cells born 1 week after testing, which were 3 weeks old at the time of the sacrifice, was higher in AI rats compared to AU rats. These interindividual differences in survival likely come from initial differences in cell proliferation. Thus, an aberrant delayed rebound in cell proliferation may also participate in memory impairment.

The homeostatic regulation of cell survival observed in AU rats (and in young rats) is consistent with the selective stabilization theory, according to which only a particular set of contacts will be selected among many others, thereby sculpting the precise circuits that are crucial for a given function (Changeux *etal*., 1976). The mechanisms underlying this selective stabilization process are unknown, but it might be hypothesized that only the adult-born cells which are successfully connected, both in terms of efferent output and afferent input, are the ones rescued by activity-dependent stimuli generated in the course of learning. The other fundamental question which remains to be answered is what is the function of these surviving neurons?

### Biological mechanism involved in age-related decline in spatial memory and neurogenesis in old rats

We have studied the role of the HPA axis in the existence of such phenotypic interindividual variations. Indeed, its up-regulation is involved in the appearance of age-related disorders (Landfield *etal*., 1978, 1981; Issa *etal*., 1990; Sapolsky, 1992). Furthermore, corticosterone inhibits neurogenesis in the aged brain (see pp. 8–9). Thus, the hypothesis at work was that excessive levels of corticosterone throughout the life of the animal would favor the emergence of memory deficits (pathological aging) by reducing neurogenesis. In favor of this hypothesis, we found that the magnitude of HPA axis activity in old animals was correlated with their level of hippocampal neurogenesis and memory ability. Indeed, animals with the heaviest adrenal glands, indicative of chronic HPA axis hyperactivity, exhibited the worst performance in the water maze and the lowest number of proliferating cells or 3-week-old surviving cells (Montaron *etal*., 2006). This indicates that hyperactivity of the HPA axis produces spatial memory deficits by decreasing hippocampal neurogenesis. In order to strengthen this hypothesis, the secretion of corticosterone was reduced from midlife onward by adrenalectomy, and its effect on neurogenesis and spatial memory ability in old rats was analyzed. It was found that lowering corticosterone secretion from midlife onward reduced the decline in neurogenesis observed in old rats and prevented age-related memory disorders (Montaron *etal*., 2006). These results demonstrate that exposure to high levels of corticosterone throughout life is responsible for age-related memory disorders and the age-related decline in neurogenesis. It remains to be determined whether other biological factors described to modulate neurogenesis in the aged brain (see Table 3) could be useful in preventing or curing the development of memory disorders during the course of aging.

**Table 3:** Aging of the neurogenic microenvironment and its effects on neurogenesis

	Evolution with aging		Effect on neurogenesis in aged brain	
Corticosteroids	Increase basal levelProlonged stress-induced secretion	(Sapolsky, 1992)	Acute ADX: increase of cell proliferation in the DG	(Cameron & McKay, 1999)
	Increased expression of GR by precursors	(Garcia *etal*., 2004a)	Long-term ADX: increase of cell proliferation in the DG	
	Expression of MR by precursors		Inverse correlation between adrenal glands’ weight and proliferation or number of new neurons in the DG	(Montaron *etal*., 1999) (Montaron *etal*., 2006) (Montaron *etal*., 2006)
Neurosteroids			Acute Preg-S icv infusion: increase of cell proliferation in the DG	(Mayo *etal*., 2003)
Glutamate			NMDA-R antagonist ip injection: increase of the number of radial glia-like cells, proliferating cells and new neurons in the DG	(Nacher *etal*., 2003)
EGF signaling	Decrease of EGF-R expression in the SVZ (non-studied in DG)	(Enwere *etal*., 2004)	HB-EGF icv infusion: (3 days): increase of cell proliferation in the DG and the SVZ	(Jin *etal*., 2003a) (Enwere *etal*., 2004)
	Decrease of TGFα expression in the SVZ (non-studied in DG)		EGF icv infusion (3 days): increase of cell proliferation in the SVZ	
IGF-I	Decrease of IGF-I concentration Decrease of IGF-I receptor expression	(Sonntag *etal*., 1997) (Sonntag *etal*., 1999) (Lai *etal*., 2000) (Shetty *etal*., 2005)	IGF-I icv infusion (14 days): increase of cell proliferation in the DG	(Lichtenwalner *etal*., 2001)
FGF-2	Decrease of hippocampal concentration of FGF-2 Decrease of FGFR-2 in the DG, the SVZ, the RMS, and the OB	(Shetty *etal*., 2005) (Chadashvili & Peterson, 2006)	FGF-2 icv infusion (3 days): strong increase of cell proliferation in the aged DG and the SVZ	(Jin *etal*., 2003a)
			FGF-2 icv infusion (2 weeks): increase of both cell proliferation and dendritic growth in middle-aged DG	(Rai *etal*., 2007)
Vasculature and VEGF	Decrease of cerebral microvasculature (especially marked in DG)	(Riddle *etal*., 2003) (Hattiangady & Shetty, 2008)	?	
	Decrease of microvascular plasticity	(Sonntag *etal*., 1997)		
	Increase of the distance between precursors and blood vesselsReduced VEGF synthesis	(Shetty *etal*., 2005) (Hattiangady & Shetty, 2008)		
Cell cycle regulators	Increase of p16^INK4a^ expression (undetectable in young animals)	(Molofsky *etal*., 2006)	Bmi-1 KO mice′: premature senescence of NSC and decrease of proliferation in SVZ, the phenotype is rescued by p16^INK4a^ or p19^Arf^ deletion	(Molofsky *etal*., 2005) (Bruggeman *etal*., 2005)
		P16^INK4a^ KO mice: proliferation is increased in SVZ but not DG of aged mice	(Molofsky *etal*., 2006)

DG, dentate gyrus; EGF, epidermal growth factor; FGF, fibroblast growth factor; IGF-I, insulin-like growth factor-I; icv, intracerebroventricular; OB, olfactory bulb; Preg-S, pregnenolone sulfate; RMS, rostral migratory stream; SVZ, subventricular zone; VEGF, vascular endothelial growth factor.

### Predictive model of aging

The next question that we addressed was whether these interindividual variations in the functional expression of neurogenesis could be predicted early in life. We took advantage of natural individual differences in the activity of the HPA axis in young adult animals, which are associated with a behavioral reactivity trait, and determined whether they could predict the extent of age-induced memory impairments. Indeed, rats that are high-behavioral responders to stress (HRs) exhibit prolonged corticosterone secretion in response to stress when they are young, premature aging of the HPA axis, and an increased propensity to develop age-related memory deficits in comparison to rats that are low-behavioral responders to novelty (LRs) (Dellu *etal*., 1994, 1996). By comparing these two groups of animals that spontaneously differ in their behavioral reactivity to novelty, it was found that cell proliferation, cell survival, and consequently neurogenesis were higher in LRs in comparison to HRs (Lemaire *etal*., 1999). Because behavioral and neuroendocrinological reactivity in youth has been demonstrated to be predictive of spatial memory impairments later in life, these individual differences in neurogenesis may account, at least in part, for individual differences in memory abilities in old age. In other words, the subjects starting off with impaired neurogenesis (HRs) are predisposed to the development of age-related memory disorders. A longitudinal study, however, is necessary to verify this prediction. This raises the issue of whether boosting neurogenesis in the young HRs by exposing them to enriched lifestyle conditions (see below) will prevent the appearance of memory deficits when they reach senescence.

### Risk factor of pathological aging: early deleterious life events

The interindividual differences that we observed may result from both genetic and environmental influences that predispose the individual (vulnerable phenotype) to develop memory disorders. Given the role of stress and corticosterone in pathological aging, a particular emphasis was given to early environmental experiences such as prenatal stress. Prenatal stress is known to significantly affect the development of the brain and the organization of behavior. In particular, prenatal stress impairs memory processes, but the underlying mechanisms are unknown.

We tested the hypothesis that prenatal-stress-induced memory deficits are related to impaired neurogenesis. By comparing juvenile (28-day-old), adult (3-month-old), middle-aged (10-month-old), and old (22-month-old) animals, it was found that prenatal stress cut cell proliferation in half over a lifetime. In fact, there was a premature decline of cell proliferation with increasing age (Lemaire *etal*., 2000). *Cell survival* and *cell phenotype*, in contrast, were not influenced by prenatal stress, at least when examined in adulthood (Lemaire *etal*., 2000, 2006). As a consequence, the number of adult-born neurons was reduced in prenatally stressed rats. Increased activity of the HPA axis following prenatal stress could explain the curtailed neurogenesis. Indeed, prenatal stress increases HPA activity as assessed by adrenal mass (Lemaire *etal*., 2000), and this effect is blocked by postnatal handling, a manipulation that counteracts the down-regulation of neurogenesis induced by prenatal stress (Lemaire *etal*., 2006). These results do not exclude the possibility that other mechanisms which remain to be elucidated mediate the deleterious effect of prenatal stress on neurogenesis.

Structural hippocampal defects resulting from prenatal stress were associated with impairment in spatial memory and in learning-induced regulation of some aspects of neurogenesis. When tested at 4 months of age, a significant difference in the rate of acquisition was observed between control and prenatally stressed rats. The latter did not reach an asymptotic level of performance. This impairment was associated with a disruption of learning-induced cell proliferation. These results are in agreement with the observation that, in young and aged rats, cell proliferation is increased only when the task is mastered (Döbrössy *etal*., 2003).

In summary, early life stress has long-lasting, deleterious effects on hippocampal neurogenesis, producing impairment in hippocampal-related spatial tasks and blocking the learning-induced increase in cell proliferation. More importantly, the neurobiological consequences of prenatal stress on hippocampal neurogenesis can be reversed with a form of postnatal environmental stimulation, therefore suggesting that pathological aging could be prevented.

### Preventing pathological aging: positive life events

An intellectually and a physically active life is known to protect from memory impairment during the course of ‘normal’ aging or in neurodegenerative disorders (Laurin *etal*., 2001; Vaillant & Mukamal, 2001; Le Carret *etal*., 2005; Hattori *etal*., 2007). Therefore, it is of importance to determine whether age-related decline in memory and neurogenesis can be reversed by stimulating life events.

#### Enriched environment

Exposure of young adult rodents to a complex enriched environment increases hippocampal neurogenesis by promoting neuron survival (Kempermann *etal*., 1997; Nilsson *etal*., 1999; Auvergne *etal*., 2002; Brown *etal*., 2003; Ueda *etal*., 2005; Rossi *etal*., 2006; Hattori *etal*., 2007; Tashiro *etal*., 2007) and, in some cases, by increasing cell proliferation (Kempermann *etal*., 1998a). At the same time, these conditions improve some aspects of spatial learning (Kempermann *etal*., 1997; Nilsson *etal*., 1999).

In the aging brain, short-term (40-day-long) (Kempermann *etal*., 1998b) or long-term (10 months starting at 10 months old) (Kempermann *etal*., 2002) exposures to an enriched environment improve neurogenesis in senescent subjects. This effect has been associated with changes in cell survival, neuronal differentiation (Kempermann *etal*., 1998b), and/or cell proliferation, although this latter effect did not reach statistical significance (Kempermann *etal*., 2002). From a functional point of view, a slight improvement in spatial performances was observed when animals were tested in the water maze after only 40 days of enriched environment (Kempermann *etal*., 1998b), while a longer exposure, from 10 months to 20 months of age, yielded a stronger improvement of enriched mice scores (Kempermann *etal*., 2002).

#### Physical activity

Voluntary access to a running wheel has been shown to be one of the components that may lead to an increase in neurogenesis when living in an enriched environment. However, besides promoting the survival of the newborn cells and their neuronal differentiation, exercise has been shown to also increase cell proliferation in young adults (van Praag *etal*., 1999a,b).

In 19-month-old mice, 45 days of unlimited access to a running wheel restores the subsequent number of surviving newly born cells to a level corresponding to 3 months old, non-running mice (van Praag *etal*., 2005). Furthermore, the percentage of cells differentiating into a neuronal phenotype shows a 2.5-fold increase. The pro-neurogenic results of exercise were also observed at both 10 months and 20 months of age after an ‘acute’ shorter exposition to the running wheel (Kronenberg *etal*., 2006). Interestingly, whereas in young animals a longer exposure to the running wheel suppresses the proliferative effect, exercise from 3 months to 9 months of age significantly reduces the age-dependent decline in cell proliferation, even if it does not maintain net neurogenesis at levels corresponding to a younger age (Kronenberg *etal*., 2006). In 14-month-old rats, a smaller yet significant enhancement of cell proliferation is also observed after short-term exposure to a treadmill (Kim *etal*., 2004). As expected, experience-induced increases in neurogenesis were associated with an improvement in spatial learning abilities (van Praag *etal*., 2005). Recently, it has been shown that maternal running and swimming cause increases in the cell genesis (Bick-Sander *etal*., 2006; Lee *etal*., 2006; Kim *etal*., 2007) and short-term memory (Lee *etal*., 2006; Kim *etal*., 2007) of the offspring. These data suggest that such ‘prenatal exercise’ may favor successful aging.

Altogether, these results show that positive life events such as exposure to a variety of new (and changing) stimuli throughout life may improve memory function by increasing neurogenesis during the course of aging. Recently, it has been shown that social isolation leading to an HPA axis hyperactivity precludes the positive effects of exercise on neurogenesis (Stranahan *etal*., 2006). This suggests that the existence of interindividual differences should be taken into account in the future as they may buffer or preclude the effects of positive life events on the aging brain.

## Conclusion

**Fig. 3:**
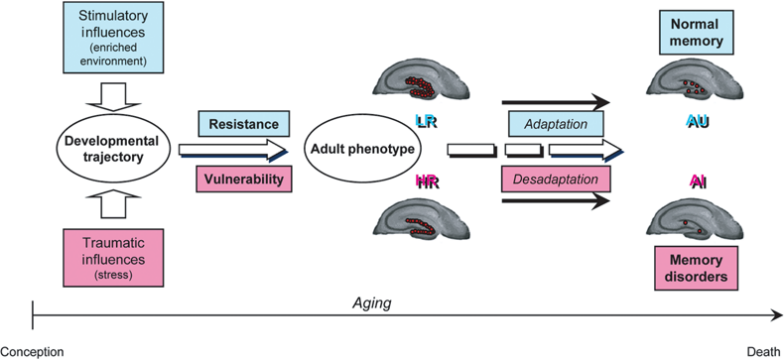
Conceptual framework highlighting the role played by hippocampal neurogenesis in environmentally induced vulnerability to the development of pathological aging. In aged rats, interindividual differences in the risk of developing age-related memory disorders are associated with individual differences in hippocampal neurogenesis. These interindividual differences can be predicted earlier in life. Animals with a low level of neurogenesis [i.e. high-behavioral responders to stress (HRs)] may not be able to adapt to environmental demands and, thus, may be more vulnerable to aging processes. On the other hand, the low-behavioral responders to novelty (LRs) starting off with a high level of neurogenesis would be resilient to the development of age-related memory disorders. These different phenotypes may result from early environmental experiences. For example, stressful events during the pre- and/or the postnatal period may lead to an HR phenotype and vulnerability to the aging processes. On the contrary, positive life events during development might favor an LR phenotype, resistant to the appearance of memory disorders.

Altogether, the results shown strongly suggest that hippocampal neurogenesis is involved in the aging of memory functions (see Fig. 3). Aging is not inescapable, and wide interindividual differences are observed among aged subjects. These differences in the risk of developing age-related memory disorders can be predicted earlier in life. Low hippocampal plasticity may render animals more vulnerable to aging processes. On the other hand, subjects starting off with a high level of neurogenesis may be resistant to the development of age-related memory disorders. These different phenotypic orientations may result from early deleterious or positive life events, which will shape the developmental trajectory of the subjects within a genetic envelope. For example, prenatal stress affects neurogenesis in pathological ways throughout life and precipitates age-related memory impairments. On the contrary, positive life events such as perinatal enrichment may prevent the appearance of an age-related decline in neurogenesis and memory abilities. Given that hippocampal neurogenesis plays a pivotal role in environmentally induced vulnerability to the development of pathological aging, a better understanding of the mechanisms that regulate neurogenesis during aging is required. Indeed, the challenge for future research will be to develop therapeutic strategies aimed at first stimulating plasticity in the aged brain and preventing the alteration of memory function that may appear in some individuals.

## References

[b1] Aberg MA, Aberg ND, Hedbacker H, Oscarsson J, Eriksson PS (2000). Peripheral infusion of IGF-I selectively induces neurogenesis in the adult rat hippocampus. J. Neurosci.

[b2] Abrous DN, Wojtowicz JM (2008). Neurogenesis and Hippocampal Memory System. Adult neurogenesis.

[b3] Abrous DN, Koehl M, Le Moal M (2005). Adult neurogenesis: from precursors to network and physiology. Physiol. Rev.

[b4] Aimone JB, Wiles J, Gage FH (2006). Potential role for adult neurogenesis in the encoding of time in new memories. Nat. Neurosci.

[b5] Alonso G (2001). Proliferation of progenitor cells in the adult rat brain correlates with the presence of vimentin-expressing astrocytes. Glia.

[b6] Ambrogini P, Orsini L, Mancini C, Ferri P, Barbanti I, Cuppini R (2002). Persistently high corticosterone levels but not normal circadian fluctuations of the hormone affect cell proliferation in the adult rat dentate gyrus. Neuroendocrinology.

[b7] Anderson MF, Aberg MA, Nilsson M, Eriksson PS (2002). Insulin-like growth factor-I and neurogenesis in the adult mammalian brain. Brain Res. Dev. Brain Res.

[b8] Anlar B, Sullivan KA, Feldman EL (1999). Insulin-like growth factor-I and central nervous system development. Horm. Metab. Res.

[b9] Auvergne R, Lere C, El Bahh B, Arthaud S, Lespinet V, Rougier A, Le Gal LS (2002). Delayed kindling epileptogenesis and increased neurogenesis in adult rats housed in an enriched environment. Brain Res.

[b10] Baker J, Liu JP, Robertson EJ, Efstratiadis A (1993). Role of insulin-like growth factors in embryonic and postnatal growth. Cell.

[b11] Baulieu EE, Robel P (1997). Neurosteroids: a new brain function. J. Steroid Biochem. Mol. Biol.

[b12] Bick-Sander A, Steiner B, Wolf SA, Babu H, Kempermann G (2006). Running in pregnancy transiently increases postnatal hippocampal neurogenesis in the offspring. Proc. Natl. Acad. Sci. USA.

[b13] Bizon JL, Gallagher M (2003). Production of new cells in the rat dentate gyrus over the lifespan: relation to cognitive decline. Eur. J. Neurosci.

[b14] Bizon JL, Lee HJ, Gallagher M (2004). Neurogenesis in a rat model of age-related cognitive decline. Aging Cell.

[b15] Bondolfi L, Ermini F, Long JM, Ingram DK, Jucker M (2004). Impact of age and caloric restriction on neurogenesis in the dentate gyrus of C57BL/6 mice. Neurobiol. Aging.

[b16] Bondy CA (1991). Transient *IGF-I* gene expression during the maturation of functionally related central projection neurons. J. Neurosci.

[b17] Bovetti S, Hsieh YC, Bovolin P, Perroteau I, Kazunori T, Puche AC (2007). Blood vessels form a scaffold for neuroblast migration in the adult olfactory bulb. J. Neurosci.

[b18] Brown J, Cooper-Kuhn CM, Kempermann G, van Praag H, Winkler J, Gage FH, Kuhn HG (2003). Enriched environment and physical activity stimulate hippocampal but not olfactory bulb neurogenesis. Eur. J. Neurosci.

[b19] Bruggeman SW, Valk-Lingbeek ME, van der Stoop PP, Jacobs JJ, Kieboom K, Tanger E, Hulsman D, Leung C, Arsenijevic Y, Marino S, van Lohuizen M (2005). Ink4a and Arf differentially affect cell proliferation and neural stem cell self-renewal in Bmi1-deficient mice. Genes Dev.

[b20] Brunson KL, Baram TZ, Bender RA (2005). Hippocampal neurogenesis is not enhanced by lifelong reduction of glucocorticoid levels. Hippocampus.

[b21] Burke SN, Barnes CA (2006). Neural plasticity in the ageing brain. Nat. Rev. Neurosci.

[b22] Cameron HA, Gould E (1994). Adult neurogenesis is regulated by adrenal steroids in the dentate gyrus. Neuroscience.

[b23] Cameron HA, McKay RD (1999). Restoring production of hippocampal neurons in old age. Nat. Neurosci.

[b24] Cameron HA, McEwen BS, Gould E (1995). Regulation of adult neurogenesis by excitatory input and NMDA receptor activation in the dentate gyrus. J. Neurosci.

[b25] Cameron HA, Tanapat P, Gould E (1998). Adrenal steroids and *N*-methyl-d-aspartate receptor activation regulate neurogenesis in the dentate gyrus of adult rats through a common pathway. Neuroscience.

[b26] Cao L, Jiao X, Zuzga DS, Liu Y, Fong DM, Young D, During MJ (2004). VEGF links hippocampal activity with neurogenesis, learning and memory. Nat. Genet.

[b27] Carlen M, Cassidy RM, Brismar H, Smith GA, Enquist LW, Frisen J (2002). Functional integration of adult-born neurons. Curr. Biol.

[b28] Chadashvili T, Peterson DA (2006). Cytoarchitecture of fibroblast growth factor receptor 2 (FGFR-2) immunoreactivity in astrocytes of neurogenic and non-neurogenic regions of the young adult and aged rat brain. J. Comp. Neurol.

[b29] Changeux JP, Benedetti L, Bourgeois JP, Brisson A, Cartaud J, Devaux P, Grunhagen H, Moreau M, Popot JL, Sobel A, Weber M (1976). Some structural properties of the cholinergic receptor protein in its membrane environmental relevant to its function as a pharmacological receptor. Cold Spring Harb. Symp. Quant. Biol.

[b30] Christie BR, Cameron HA (2006). Neurogenesis in the adult hippocampus. Hippocampus.

[b31] Cuppini R, Bucherelli C, Ambrogini P, Ciuffoli S, Orsini L, Ferri P, Baldi E (2006). Age-related naturally occuring depression of hippocampal neurogenesis does not affect trace fear conditioning. Hippocampus.

[b32] Czeh B, Michaelis T, Watanabe T, Frahm J, de Biurrun G, van Kampen M, Bartolomucci A, Fuchs E (2001). Stress-induced changes in cerebral metabolites, hippocampal volume, and cell proliferation are prevented by antidepressant treatment with tianeptine. Proc. Natl. Acad. Sci. USA.

[b33] Deisseroth K, Singla S, Toda H, Monje M, Palmer TD, Malenka RC (2004). Excitation-neurogenesis coupling in adult neural stem/progenitor cells. Neuron.

[b34] Dellu F, Mayo W, Vallee M, Le Moal M, Simon H (1994). Reactivity to novelty during youth as a predictive factor of cognitive impairment in the elderly: a longitudinal study in rats. Brain Res.

[b35] Dellu F, Mayo W, Vallee M, Maccari S, Piazza PV, Le Moal M, Simon H (1996). Behavioral reactivity to novelty during youth as a predictive factor of stress-induced corticosterone secretion in the elderly – a life-span study in rats. Psychoneuroendocrinology.

[b36] Dobrossy MD, Drapeau E, Aurousseau C, Le Moal M, Piazza PV, Abrous DN (2003). Differential effects of learning on neurogenesis: learning increases or decreases the number of newly born cells depending on their birth date. Mol. Psychiatry.

[b37] Doetsch F, Caille I, Lim DA, Garcia-Verdugo JM, Alvarez-Buylla A (1999a). Subventricular zone astrocytes are neural stem cells in the adult mammalian brain. Cell.

[b38] Doetsch F, Garcia-Verdugo JM, Alvarez-Buylla A (1999b). Regeneration of a germinal layer in the adult mammalian brain. Proc. Natl. Acad. Sci. USA.

[b39] Drapeau E, Montaron MF, Aguerre S, Abrous DN (2007). Learning-induced survival of new neurons depends on the cognitive status of aged rats. J. Neurosci.

[b40] Driscoll I, Howard SR, Stone JC, Monfils MH, Tomanek B, Brooks WM, Sutherland RJ (2006). The aging hippocampus: a multi-level analysis in the rat. Neuroscience.

[b41] Du Y, Dreyfus CF (2002). Oligodendrocytes as providers of growth factors. J. Neurosci. Res.

[b42] Dupret D, Fabre A, Dobrossy MD, Panatier A, Rodriguez JJ, Lamarque S, Lemaire V, Oliet SH, Piazza PV, Abrous DN (2007). Spatial learning depends on both the addition and removal of new hippocampal neurons. PLoS Biol.

[b43] van Eekelen JA, Jiang W, De Kloet ER, Bohn MC (1988). Distribution of the mineralocorticoid and the glucocorticoid receptor mRNAs in the rat hippocampus. J. Neurosci. Res.

[b44] van Eekelen JA, Rots NY, Sutanto W, Oitzl MS, De Kloet ER (1991). Brain corticosteroid receptor gene expression and neuroendocrine dynamics during aging. J. Steroid Biochem. Mol. Biol.

[b45] Encinas JM, Vaahtokari A, Enikolopov G (2006). Fluoxetine targets early progenitor cells in the adult brain. Proc. Natl. Acad. Sci. USA.

[b46] Enwere E, Shingo T, Gregg C, Fujikawa H, Ohta S, Weiss S (2004). Aging results in reduced epidermal growth factor receptor signaling, diminished olfactory neurogenesis, and deficits in fine olfactory discrimination. J. Neurosci.

[b47] Fabel K, Fabel K, Tam B, Kaufer D, Baiker A, Simmons N, Kuo CJ, Palmer TD (2003). VEGF is necessary for exercise-induced adult hippocampal neurogenesis. Eur. J. Neurosci.

[b48] Fernandez-Galaz MC, Morschl E, Chowen JA, Torres-Aleman I, Naftolin F, Garcia-Segura LM (1997). Role of astroglia and insulin-like growth factor-I in gonadal hormone-dependent synaptic plasticity. Brain Res. Bull.

[b49] Gage FH, Chen KS, Buzsaki G, Armstrong D (1988). Experimental approaches to age-related cognitive impairments. Neurobiol. Aging.

[b50] Gallagher M, Burwell RD, Kodsi MH, McKinney M, Southerland S, Vella-Rountree L, Lewis MH (1990). Markers for biogenic amines in the aged rat brain: relationship to decline in spatial learning ability. Neurobiol. Aging.

[b51] Gallagher M, Burwell R, Burchinal M (1993). Severity of spatial learning impairment in aging: development of a learning index for performance in the Morris water maze. Behav. Neurosci.

[b52] Garcia A, Steiner B, Kronenberg G, Bick-Sander A, Kempermann G (2004a). Age-dependent expression of glucocorticoid- and mineralocorticoid receptors on neural precursor cell populations in the adult murine hippocampus. Aging Cell.

[b53] Garcia AD, Doan NB, Imura T, Bush TG, Sofroniew MV (2004b). GFAP-expressing progenitors are the principal source of constitutive neurogenesis in adult mouse forebrain. Nat. Neurosci.

[b54] Ge S, Pradhan DA, Ming GL, Song H (2007). GABA sets the tempo for activity-dependent adult neurogenesis. Trends Neurosci.

[b55] Gould E, Tanapat P (1999). Stress and hippocampal neurogenesis. Biol. Psychiatry.

[b56] Gould E, Cameron HA, Daniels DC, Woolley CS, McEwen BS (1992). Adrenal hormones suppress cell division in the adult rat dentate gyrus. J. Neurosci.

[b57] Gould E, McEwen BS, Tanapat P, Galea LA, Fuchs E (1997). Neurogenesis in the dentate gyrus of the adult tree shrew is regulated by psychosocial stress and NMDA receptor activation. J. Neurosci.

[b58] Gould E, Tanapat P, McEwen BS, Flugge G, Fuchs E (1998). Proliferation of granule cell precursors in the dentate gyrus of adult monkeys is diminished by stress. Proc. Natl. Acad. Sci. USA.

[b59] Gould E, Beylin A, Tanapat P, Reeves A, Shors TJ (1999). Learning enhances adult neurogenesis in the hippocampal formation. Nat. Neurosci.

[b60] Grady CL, Craik FI (2000). Changes in memory processing with age. Curr. Opin. Neurobiol.

[b61] Greenberg DA, Jin K (2004). Experiencing VEGF. Nat. Genet.

[b62] Gross CG (2000). Neurogenesis in the adult brain: death of a dogma. Nat. Rev. Neurosci.

[b63] Hastings NB, Gould E (1999). Rapid extension of axons into the CA3 region by adult-generated granule cells. J. Comp. Neurol.

[b64] Hattiangady B, Shetty AK (2008). Aging does not alter the number or phenotype of putative stem/progenitor cells in the neurogenic region of the hippocampus. Neurobiol. Aging.

[b65] Hattori S, Hashimoto R, Miyakawa T, Yamanaka H, Maeno H, Wada K, Kunugi H (2007). Enriched environments influence depression-related behavior in adult mice and the survival of newborn cells in their hippocampi. Behav. Brain Res.

[b66] Heine VM, Maslam S, Joels M, Lucassen PJ (2004a). Prominent decline of newborn cell proliferation, differentiation, and apoptosis in the aging dentate gyrus, in absence of an age-related hypothalamus–pituitary–adrenal axis activation. Neurobiol. Aging.

[b67] Heine VM, Maslam S, Zareno J, Joels M, Lucassen PJ (2004b). Suppressed proliferation and apoptotic changes in the rat dentate gyrus after acute and chronic stress are reversible. Eur. J. Neurosci.

[b68] Heine VM, Zareno J, Maslam S, Joels M, Lucassen PJ (2005). Chronic stress in the adult dentate gyrus reduces cell proliferation near the vasculature and VEGF and Flk-1 protein expression. Eur. J. Neurosci.

[b69] Huang L, Bittman EL (2002). Olfactory bulb cells generated in adult male golden hamsters are specifically activated by exposure to estrous females. Horm. Behav.

[b70] Hulshoff Pol HE, Hijman R, Baare WF, van Eekelen S, van Ree JM (2000). Odor discrimination and task duration in young and older adults. Chem. Senses.

[b71] Issa AM, Rowe W, Gauthier S, Meaney MJ (1990). Hypothalamic–pituitary–adrenal activity in aged, cognitively impaired and cognitively unimpaired rats. J. Neurosci.

[b72] Jacobs JJ, Kieboom K, Marino S, DePinho RA, van Lohuizen M (1999). The oncogene and Polycomb-group gene bmi-1 regulates cell proliferation and senescence through the ink4a locus. Nature.

[b73] Jessberger S, Kempermann G (2003). Adult-born hippocampal neurons mature into activity-dependent responsiveness. Eur. J. Neurosci.

[b74] Jin K, Mao XO, Sun Y, Xie L, Jin L, Nishi E, Klagsbrun M, Greenberg DA (2002a). Heparin-binding epidermal growth factor-like growth factor: hypoxia-inducible expression *in vitro* and stimulation of neurogenesis *in vitro* and *in vivo*. J. Neurosci.

[b75] Jin K, Zhu Y, Sun Y, Mao XO, Xie L, Greenberg DA (2002b). Vascular endothelial growth factor (VEGF) stimulates neurogenesis *in vitro* and *in vivo*. Proc. Natl. Acad. Sci. USA.

[b76] Jin K, Sun Y, Xie L, Batteur S, Mao XO, Smelick C, Logvinova A, Greenberg DA (2003a). Neurogenesis and aging: FGF-2 and HB-EGF restore neurogenesis in hippocampus and subventricular zone of aged mice. Aging Cell.

[b77] Jin K, Xie L, Childs J, Sun Y, Mao XO, Logvinova A, Greenberg DA (2003b). Cerebral neurogenesis is induced by intranasal administration of growth factors. Ann. Neurol.

[b78] Kaneda H, Maeshima K, Goto N, Kobayakawa T, Ayabe-Kanamura S, Saito S (2000). Decline in taste and odor discrimination abilities with age, and relationship between gustation and olfaction. Chem. Senses.

[b79] Karishma KK, Herbert J (2002). Dehydroepiandrosterone (DHEA) stimulates neurogenesis in the hippocampus of the rat, promotes survival of newly formed neurons and prevents corticosterone-induced suppression. Eur. J. Neurosci.

[b80] Kee N, Sivalingam S, Boonstra R, Wojtowicz JM (2002). The utility of Ki-67 and BrdU as proliferative markers of adult neurogenesis. J. Neurosci. Methods.

[b81] Kee N, Teixeira CM, Wang AH, Frankland PW (2007). Preferential incorporation of adult-generated granule cells into spatial memory networks in the dentate gyrus. Nat. Neurosci.

[b82] Kempermann G, Kuhn HG, Gage FH (1997). More hippocampal neurons in adult mice living in an enriched environment. Nature.

[b83] Kempermann G, Brandon EP, Gage FH (1998a). Environmental stimulation of 129/SvJ mice causes increased cell proliferation and neurogenesis in the adult dentate gyrus. Curr. Biol.

[b84] Kempermann G, Kuhn HG, Gage FH (1998b). Experience-induced neurogenesis in the senescent dentate gyrus. J. Neurosci.

[b85] Kempermann G, Gast D, Gage FH (2002). Neuroplasticity in old age: sustained fivefold induction of hippocampal neurogenesis by long-term environmental enrichment. Ann. Neurol.

[b86] Kim YP, Kim H, Shin MS, Chang HK, Jang MH, Shin MC, Lee SJ, Lee HH, Yoon JH, Jeong IG, Kim CJ (2004). Age-dependence of the effect of treadmill exercise on cell proliferation in the dentate gyrus of rats. Neurosci. Lett.

[b87] Kim H, Lee SH, Kim SS, Yoo JH, Kim CJ (2007). The influence of maternal treadmill running during pregnancy on short-term memory and hippocampal cell survival in rat pups. Int. J. Dev. Neurosci.

[b88] Krishnamurthy J, Torrice C, Ramsey MR, Kovalev GI, Al Regaiey K, Su L, Sharpless NE (2004). Ink4a/Arf expression is a biomarker of aging. J. Clin. Invest.

[b89] Kronenberg G, Bick-Sander A, Bunk E, Wolf C, Ehninger D, Kempermann G (2006). Physical exercise prevents age-related decline in precursor cell activity in the mouse dentate gyrus. Neurobiol. Aging.

[b90] Kuhn HG, Dickinson-Anson H, Gage FH (1996). Neurogenesis in the dentate gyrus of the adult rat: age-related decrease of neuronal progenitor proliferation. J. Neurosci.

[b91] Kuhn HG, Winkler J, Kempermann G, Thal LJ, Gage FH (1997). Epidermal growth factor and fibroblast growth factor-2 have different effects on neural progenitors in the adult rat brain. J. Neurosci.

[b92] Lai M, Hibberd CJ, Gluckman PD, Seckl JR (2000). Reduced expression of insulin-like growth factor 1 messenger RNA in the hippocampus of aged rats. Neurosci. Lett.

[b93] Landfield PW, Waymire JC, Lynch G (1978). Hippocampal aging and adrenocorticoids: quantitative correlations. Science.

[b94] Landfield PW, Baskin RK, Pitler TA (1981). Brain aging correlates: retardation by hormonal–pharmacological treatments. Science.

[b95] Laurin D, Verreault R, Lindsay J, MacPherson K, Rockwood K (2001). Physical activity and risk of cognitive impairment and dementia in elderly persons. Arch. Neurol.

[b96] Lazar LM, Blum M (1992). Regional distribution and developmental expression of epidermal growth factor and transforming growth factor-alpha mRNA in mouse brain by a quantitative nuclease protection assay. J. Neurosci.

[b97] Le Carret N, Auriacombe S, Letenneur L, Bergua V, Dartigues JF, Fabrigoule C (2005). Influence of education on the pattern of cognitive deterioration in AD patients: the cognitive reserve hypothesis. Brain Cogn.

[b98] Lee HH, Kim H, Lee JW, Kim YS, Yang HY, Chang HK, Lee TH, Shin MC, Lee MH, Shin MS, Park S, Baek S, Kim CJ (2006). Maternal swimming during pregnancy enhances short-term memory and neurogenesis in the hippocampus of rat pups. Brain Dev.

[b99] Lemaire V, Aurousseau C, Le Moal M, Abrous DN (1999). Behavioural trait of reactivity to novelty is related to hippocampal neurogenesis. Eur. J. Neurosci.

[b100] Lemaire V, Koehl M, Le Moal M, Abrous DN (2000). Prenatal stress produces learning deficits associated with an inhibition of neurogenesis in the hippocampus. Proc. Natl. Acad. Sci. USA.

[b101] Lemaire V, Lamarque S, Le Moal M, Piazza PV, Abrous DN (2006). Postnatal stimulation of the pups counteracts prenatal stress-induced deficits in hippocampal neurogenesis. Biol. Psychiatry.

[b102] Lessard J, Sauvageau G (2003). Bmi-1 determines the proliferative capacity of normal and leukaemic stem cells. Nature.

[b103] Leuner B, Gould E, Shors TJ (2006). Is there a link between adult neurogenesis and learning?. Hippocampus.

[b104] Lichtenwalner RJ, Forbes ME, Bennett SA, Lynch CD, Sonntag WE, Riddle DR (2001). Intracerebroventricular infusion of insulin-like growth factor-I ameliorates the age-related decline in hippocampal neurogenesis. Neuroscience.

[b105] Lledo PM, Alonso M, Grubb MS (2006). Adult neurogenesis and functional plasticity in neuronal circuits. Nat. Rev. Neurosci.

[b106] Lois C, Alvarez-Buylla A (1994). Long-distance neuronal migration in the adult mammalian brain. Science.

[b107] Luo J, Daniels SB, Lennington JB, Notti RQ, Conover JC (2006). The aging neurogenic subventricular zone. Aging Cell.

[b108] Lupien S, Lecours AR, Lussier I, Schwartz G, Nair NP, Meaney MJ (1994). Basal cortisol levels and cognitive deficits in human aging. J. Neurosci.

[b109] Luskin MB (1993). Restricted proliferation and migration of postnatally generated neurons derived from the forebrain subventricular zone. Neuron.

[b110] Magavi SS, Mitchell BD, Szentirmai O, Carter BS, Macklis JD (2005). Adult-born and preexisting olfactory granule neurons undergo distinct experience-dependent modifications of their olfactory responses *in vivo*. J. Neurosci.

[b111] Marczynski TJ (1998). GABAergic deafferentation hypothesis of brain aging and Alzheimer's disease revisited. Brain Res. Bull.

[b112] Markakis EA, Gage FH (1999). Adult-generated neurons in the dentate gyrus send axonal projections to field CA3 and are surrounded by synaptic vesicles. J. Comp. Neurol.

[b113] Markowska AL, Stone WS, Ingram DK, Reynolds J, Gold PE, Conti LH, Pontecorvo MJ, Wenk GL, Olton DS (1989). Individual differences in aging: behavioral and neurobiological correlates. Neurobiol. Aging.

[b114] Maslov AY, Barone TA, Plunkett RJ, Pruitt SC (2004). Neural stem cell detection, characterization, and age-related changes in the subventricular zone of mice. J. Neurosci.

[b115] Mayo W, George O, Darbra S, Bouyer JJ, Vallee M, Darnaudery M, Pallares M, Lemaire-Mayo V, Le Moal M, Piazza PV, Abrous N (2003). Individual differences in cognitive aging: implication of pregnenolone sulfate. Prog. Neurobiol.

[b116] McDonald HY, Wojtowicz JM (2005). Dynamics of neurogenesis in the dentate gyrus of adult rats. Neurosci. Lett.

[b117] Meaney MJ, Aitken DH, Sharma S, Viau V (1992). Basal ACTH, corticosterone and corticosterone-binding globulin levels over the diurnal cycle, and age-related changes in hippocampal type I and type II corticosteroid receptor binding capacity in young and aged, handled and nonhandled rats. Neuroendocrinology.

[b118] Merrill DA, Chiba AA, Tuszynski MH (2001). Conservation of neuronal number and size in the entorhinal cortex of behaviorally characterized aged rats. J. Comp. Neurol.

[b119] Merrill DA, Karim R, Darraq M, Chiba AA, Tuszynski MH (2003). Hippocampal cell genesis does not correlate with spatial learning ability in aged rats. J. Comp. Neurol.

[b120] Ming GL, Song H (2005). Adult neurogenesis in the mammalian central nervous system. Annu. Rev. Neurosci.

[b121] Molofsky AV, Pardal R, Iwashita T, Park IK, Clarke MF, Morrison SJ (2003). Bmi-1 dependence distinguishes neural stem cell self-renewal from progenitor proliferation. Nature.

[b122] Molofsky AV, He S, Bydon M, Morrison SJ, Pardal R (2005). Bmi-1 promotes neural stem cell self-renewal and neural development but not mouse growth and survival by repressing the p16Ink4a and p19Arf senescence pathways. Genes Dev.

[b123] Molofsky AV, Slutsky SG, Joseph NM, He S, Pardal R, Krishnamurthy J, Sharpless NE, Morrison SJ (2006). Increasing p16INK4a expression decreases forebrain progenitors and neurogenesis during ageing. Nature.

[b124] Montaron MF, Petry KG, Rodriguez JJ, Marinelli M, Aurousseau C, Rougon G, Le Moal M, Abrous DN (1999). Adrenalectomy increases neurogenesis but not PSA-NCAM expression in aged dentate gyrus. Eur. J. Neurosci.

[b125] Montaron MF, Piazza PV, Aurousseau C, Urani A, Le Moal M, Abrous DN (2003). Implication of corticosteroid receptors in the regulation of hippocampal structural plasticity. Eur. J. Neurosci.

[b126] Montaron MF, Drapeau E, Dupret D, Kitchener P, Aurousseau C, Le Moal M, Piazza PV, Abrous DN (2006). Lifelong corticosterone level determines age-related decline in neurogenesis and memory. Neurobiol. Aging.

[b127] Morris RG (2006). Elements of a neurobiological theory of hippocampal function: the role of synaptic plasticity, synaptic tagging and schemas. Eur. J. Neurosci.

[b128] Morrison RS, Keating RF, Moskal JR (1988). Basic fibroblast growth factor and epidermal growth factor exert differential trophic effects on CNS neurons. J. Neurosci. Res.

[b129] Nacher J, Rosell DR, Alonso-Llosa G, McEwen BS (2001). NMDA receptor antagonist treatment induces a long-lasting increase in the number of proliferating cells, PSA-NCAM-immunoreactive granule neurons and radial glia in the adult rat dentate gyrus. Eur. J. Neurosci.

[b130] Nacher J, Alonso-Llosa G, Rosell DR, McEwen BS (2003). NMDA receptor antagonist treatment increases the production of new neurons in the aged rat hippocampus. Neurobiol. Aging.

[b131] Nakagami Y, Saito H, Matsuki N (1997). Basic fibroblast growth factor and brain-derived neurotrophic factor promote survival and neuronal circuit formation in organotypic hippocampal culture. Jpn. J. Pharmacol.

[b132] Ni Dhuill CM, Fox GB, Pittock SJ, O’Connell AW, Murphy KJ, Regan CM (1999). Polysialylated neural cell adhesion molecule expression in the dentate gyrus of the human hippocampal formation from infancy to old age. J. Neurosci. Res.

[b133] Nilsson M, Perfilieva E, Johansson U, Orwar O, Eriksson PS (1999). Enriched environment increases neurogenesis in the adult rat dentate gyrus and improves spatial memory. J. Neurobiol.

[b134] Olariu A, Cleaver KM, Cameron HA (2007). Decreased neurogenesis in aged rats results from loss of granule cell precursors without lengthening of the cell cycle. J. Comp. Neurol.

[b135] Opanashuk LA, Mark RJ, Porter J, Damm D, Mattson MP, Seroogy KB (1999). Heparin-binding epidermal growth factor-like growth factor in hippocampus: modulation of expression by seizures and anti-excitotoxic action. J. Neurosci.

[b136] Palmer TD, Willhoite AR, Gage FH (2000). Vascular niche for adult hippocampal neurogenesis. J. Comp. Neurol.

[b137] Park IK, Qian D, Kiel M, Becker MW, Pihalja M, Weissman IL, Morrison SJ, Clarke MF (2003). Bmi-1 is required for maintenance of adult self-renewing haematopoietic stem cells. Nature.

[b138] Petit TD, Ivy O (1988). Neuroplasticity, Learning and Memory, Neurology and Neurobiology.

[b139] Petreanu L, Alvarez-Buylla A (2002). Maturation and death of adult-born olfactory bulb granule neurons: role of olfaction. J. Neurosci.

[b140] Pham K, Nacher J, Hof PR, McEwen BS (2003). Repeated restraint stress suppresses neurogenesis and induces biphasic PSA–NCAM expression in the adult rat dentate gyrus. Eur. J. Neurosci.

[b141] van Praag H, Christie BR, Sejnowski TJ, Gage FH (1999a). Running enhances neurogenesis, learning, and long-term potentiation in mice. Proc. Natl. Acad. Sci. USA.

[b142] van Praag H, Kempermann G, Gage FH (1999b). Running increases cell proliferation and neurogenesis in the adult mouse dentate gyrus. Nat. Neurosci.

[b143] van Praag H, Schinder AF, Christie BR, Toni N, Palmer TD, Gage FH (2002). Functional neurogenesis in the adult hippocampus. Nature.

[b144] van Praag H, Shubert T, Zhao C, Gage FH (2005). Exercise enhances learning and hippocampal neurogenesis in aged mice. J. Neurosci.

[b145] Rai KS, Hattiangady B, Shetty AK (2007). Enhanced production and dendritic growth of new dentate granule cells in the middle-aged hippocampus following intracerebroventricular FGF-2 infusions. Eur. J. Neurosci.

[b146] Rao MS, Hattiangady B, Abdel-Rahman A, Stanley DP, Shetty AK (2005). Newly born cells in the ageing dentate gyrus display normal migration, survival and neuronal fate choice but endure retarded early maturation. Eur. J. Neurosci.

[b147] Rao MS, Hattiangady B, Shetty AK (2006). The window and mechanisms of major age-related decline in the production of new neurons within the dentate gyrus of the hippocampus. Aging Cell.

[b148] Rapp PR, Amaral DG (1992). Individual differences in the cognitive and neurobiological consequences of normal aging. Trends Neurosci.

[b149] Rapp PR, Gallagher M (1996). Preserved neuron number in the hippocampus of aged rats with spatial learning deficits. Proc. Natl. Acad. Sci. USA.

[b150] Rapp PR, Deroche PS, Mao Y, Burwell RD (2002). Neuron number in the parahippocampal region is preserved in aged rats with spatial learning deficits. Cereb. Cortex.

[b151] Rasmussen T, Schliemann T, Sorensen JC, Zimmer J, West MJ (1996). Memory impaired aged rats: no loss of principal hippocampal and subicular neurons. Neurobiol. Aging.

[b152] Redish AD, Touretzky DS (1998). The role of the hippocampus in solving the Morris water maze. Neural Comput.

[b153] Riddle DR, Sonntag WE, Lichtenwalner RJ (2003). Microvascular plasticity in aging. Ageing Res. Rev.

[b154] Rodriguez JJ, Montaron MF, Petry KG, Aurousseau C, Marinelli M, Premier S, Rougon G, Le Moal M, Abrous DN (1998). Complex regulation of the expression of the polysialylated form of the neuronal cell adhesion molecule by glucocorticoids in the rat hippocampus. Eur. J. Neurosci.

[b155] Rosenzweig MR, Bennett EL (1996). Psychobiology of plasticity: effects of training and experience on brain and behavior. Behav. Brain Res.

[b156] Rossi C, Angelucci A, Costantin L, Braschi C, Mazzantini M, Babbini F, Fabbri ME, Tessarollo L, Maffei L, Berardi N, Caleo M (2006). Brain-derived neurotrophic factor (BDNF) is required for the enhancement of hippocampal neurogenesis following environmental enrichment. Eur. J. Neurosci.

[b157] Rotwein P, Burgess SK, Milbrandt JD, Krause JE (1988). Differential expression of insulin-like growth factor genes in rat central nervous system. Proc. Natl. Acad. Sci. USA.

[b158] Rowe WB, Blalock EM, Chen KC, Kadish I, Wang D, Barrett JE, Thibault O, Porter NM, Rose GM, Landfield PW (2007). Hippocampal expression analyses reveal selective association of immediate-early, neuroenergetic, and myelinogenic pathways with cognitive impairment in aged rats. J. Neurosci.

[b159] Sapolsky RM (1992). Do glucocorticoid concentrations rise with age in the rat?. Neurobiol. Aging.

[b160] Sapolsky RM, Romero LM, Munck AU (2000). How do glucocorticoids influence stress responses? Integrating permissive, suppressive, stimulatory, and preparative actions. Endocr. Rev.

[b161] Scharfman HE, Hen R (2007). Neuroscience. Is more neurogenesis always better?. Science.

[b162] Schlessinger AR, Cowan WM, Gottlieb DI (1975). An autoradiographic study of the time of origin and the pattern of granule cell migration in the dentate gyrus of the rat. J. Comp. Neurol.

[b163] Seaberg RM, van der Kooy D (2002). Adult rodent neurogenic regions: the ventricular subependyma contains neural stem cells, but the dentate gyrus contains restricted progenitors. J. Neurosci.

[b164] Segovia G, Yague AG, Garcia-Verdugo JM, Mora F (2006). Environmental enrichment promotes neurogenesis and changes the extracellular concentrations of glutamate and GABA in the hippocampus of aged rats. Brain Res. Bull.

[b165] Seki T, Arai Y (1995). Age-related production of new granule cells in the adult dentate gyrus. Neuroreport.

[b166] Seri B, Garcia-Verdugo JM, Collado-Morente L, McEwen BS, Alvarez-Buylla A (2004). Cell types, lineage, and architecture of the germinal zone in the adult dentate gyrus. J. Comp. Neurol.

[b167] Seroogy KB, Han VK, Lee DC (1991). Regional expression of transforming growth factor-alpha mRNA in the rat central nervous system. Neurosci. Lett.

[b168] Seroogy KB, Lundgren KH, Lee DC, Guthrie KM, Gall CM (1993). Cellular localization of transforming growth factor-alpha mRNA in rat forebrain. J. Neurochem.

[b169] Sherr CJ (2001). The INK4a/ARF network in tumour suppression. Nat. Rev. Mol. Cell Biol.

[b170] Shetty AK, Hattiangady B, Shetty GA (2005). Stem/Progenitor cell proliferation factors FGF-2, IGF-1, and VEGF exhibit early decline during the course of aging in the hippocampus: role of astrocytes. Glia.

[b171] Simon M, Czeh B, Fuchs E (2005). Age-dependent susceptibility of adult hippocampal cell proliferation to chronic psychosocial stress. Brain Res.

[b172] Sonntag WE, Lynch CD, Cooney PT, Hutchins PM (1997). Decreases in cerebral microvasculature with age are associated with the decline in growth hormone and insulin-like growth factor 1. Endocrinology.

[b173] Sonntag WE, Lynch CD, Bennett SA, Khan AS, Thornton PL, Cooney PT, Ingram RL, McShane T, Brunso-Bechtold JK (1999). Alterations in insulin-like growth factor-1 gene and protein expression and type 1 insulin-like growth factor receptors in the brains of ageing rats. Neuroscience.

[b174] Stanfield BB, Trice JE (1988). Evidence that granule cells generated in the dentate gyrus of adult rats extend axonal projections. Exp. Brain Res.

[b175] Stevens JC, Cain WS (1987). Old-age deficits in the sense of smell as gauged by thresholds, magnitude matching, and odor identification. Psychol. Aging.

[b176] Stoelzel CR, Stavnezer AJ, Denenberg VH, Ward M, Markus EJ (2002). The effects of aging and dorsal hippocampal lesions: performance on spatial and nonspatial comparable versions of the water maze. Neurobiol. Learn. Mem.

[b177] Stranahan AM, Khalil D, Gould E (2006). Social isolation delays the positive effects of running on adult neurogenesis. Nat. Neurosci.

[b178] Sun Y, Jin K, Xie L, Childs J, Mao XO, Logvinova A, Greenberg DA (2003). VEGF-induced neuroprotection, neurogenesis, and angiogenesis after focal cerebral ischemia. J. Clin. Invest.

[b179] Sun W, Winseck A, Vinsant S, Park OH, Kim H, Oppenheim RW (2004). Programmed cell death of adult-generated hippocampal neurons is mediated by the proapoptotic gene *Bax*. J. Neurosci.

[b180] Tanaka A, Watanabe Y, Kato H, Araki T (2007). Immunohistochemical changes related to ageing in the mouse hippocampus and subventricular zone. Mech. Ageing Dev.

[b181] Tashiro A, Makino H, Gage FH (2007). Experience-specific functional modification of the dentate gyrus through adult neurogenesis: a critical period during an immature stage. J. Neurosci.

[b182] Taupin P (2007). BrdU immunohistochemistry for studying adult neurogenesis: paradigms, pitfalls, limitations, and validation. Brain Res. Rev.

[b183] Thomas RM, Hotsenpiller G, Peterson DA (2007). Acute psychosocial stress reduces cell survival in adult hippocampal neurogenesis without altering proliferation. J. Neurosci.

[b184] Toni N, Teng EM, Bushong EA, Aimone JB, Zhao C, Consiglio A, van Praag H, Martone ME, Ellisman MH, Gage FH (2007). Synapse formation on neurons born in the adult hippocampus. Nat. Neurosci.

[b185] Trejo JL, Carro E, Torres-Aleman I (2001). Circulating insulin-like growth factor I mediates exercise-induced increases in the number of new neurons in the adult hippocampus. J. Neurosci.

[b186] Tropepe V, Craig CG, Morshead CM, van der Kooy D (1997). Transforming growth factor-alpha null and senescent mice show decreased neural progenitor cell proliferation in the forebrain subependyma. J. Neurosci.

[b187] Ueda S, Sakakibara S, Yoshimoto K (2005). Effect of long-lasting serotonin depletion on environmental enrichment-induced neurogenesis in adult rat hippocampus and spatial learning. Neuroscience.

[b188] Vaccarino FM, Schwartz ML, Raballo R, Nilsen J, Rhee J, Zhou M, Doetschman T, Coffin JD, Wyland JJ, Hung YT (1999). Changes in cerebral cortex size are governed by fibroblast growth factor during embryogenesis. Nat. Neurosci.

[b189] Vaillant GE, Mukamal K (2001). Successful aging. Am. J. Psychiatry.

[b190] Vicario-Abejon C, Johe KK, Hazel TG, Collazo D, McKay RD (1995). Functions of basic fibroblast growth factor and neurotrophins in the differentiation of hippocampal neurons. Neuron.

[b191] Vizi ES, Kiss JP (1998). Neurochemistry and pharmacology of the major hippocampal transmitter systems: synaptic and nonsynaptic interactions. Hippocampus.

[b192] Wagner JP, Black IB, DiCicco-Bloom E (1999). Stimulation of neonatal and adult brain neurogenesis by subcutaneous injection of basic fibroblast growth factor. J. Neurosci.

[b193] Walicke P, Cowan WM, Ueno N, Baird A, Guillemin R (1986). Fibroblast growth factor promotes survival of dissociated hippocampal neurons and enhances neurite extension. Proc. Natl. Acad. Sci. USA.

[b194] Wati H, Kudo K, Qiao C, Kuroki T, Kanba S (2006). A decreased survival of proliferated cells in the hippocampus is associated with a decline in spatial memory in aged rats. Neurosci Lett.

[b195] West MJ (1993). Regionally specific loss of neurons in the aging human hippocampus. Neurobiol. Aging.

[b196] West MJ, Coleman PD, Flood DG, Troncoso JC (1994). Differences in the pattern of hippocampal neuronal loss in normal ageing and Alzheimer's disease. Lancet.

[b197] Wilcox JN, Derynck R (1988). Localization of cells synthesizing transforming growth factor-alpha mRNA in the mouse brain. J. Neurosci.

[b198] Winner B, Cooper-Kuhn CM, Aigner R, Winkler J, Kuhn HG (2002). Long-term survival and cell death of newly generated neurons in the adult rat olfactory bulb. Eur. J. Neurosci.

[b199] Wojtowicz JM, Kee N (2006). BrdU assay for neurogenesis in rodents. Nat. Protoc.

[b200] Wong EY, Herbert J (2005). Roles of mineralocorticoid and glucocorticoid receptors in the regulation of progenitor proliferation in the adult hippocampus. Eur. J. Neurosci.

[b201] Yoshimura S, Takagi Y, Harada J, Teramoto T, Thomas SS, Waeber C, Bakowska JC, Breakefield XO, Moskowitz MA (2001). FGF-2 regulation of neurogenesis in adult hippocampus after brain injury. Proc. Natl. Acad. Sci. USA.

[b202] Zhang RL, Zhang Z, Zhang L, Wang Y, Zhang C, Chopp M (2006). Delayed treatment with sildenafil enhances neurogenesis and improves functional recovery in aged rats after focal cerebral ischemia. J. Neurosci. Res.

[b203] Zindy F, Quelle DE, Roussel MF, Sherr CJ (1997). Expression of the p16INK4a tumor suppressor versus other INK4 family members during mouse development and aging. Oncogene.

